# Validation of novel naturalistic limb movement stimuli for studying biological motion perception in adults

**DOI:** 10.3389/fnhum.2026.1754368

**Published:** 2026-05-07

**Authors:** Maria Koriakina, Ioannis Ntoumanis, Evgeny Blagovechtchenski, Ekaterina Pomelova, Tatiana Ledneva, Olga Agranovich, Isak B. Blank, Andriy Myachykov, Anna Shestakova

**Affiliations:** 1 Affective Psychophysiology Laboratory, Institute of Health Psychology, HSE, Saint-Petersburg, Russia; 2 Centre for Cognition and Decision Making, Institute for Cognitive Neuroscience, HSE University, Moscow, Russia; 3 Cognitive Health and Intelligence Centre, Institute for Cognitive Neuroscience, HSE University, Moscow, Russia; 4 H. Turner National Medical Research Center for Сhildren's Orthopedics and Trauma Surgery of the Ministry of Health of the Russian Federation, Saint-Petersburg, Russia; 5 Center for Cognitive and Brain Sciences, University of Macau, Taipa, Macau SAR, China

**Keywords:** action observation, affordances, biological motionperception, electroencephalography (EEG), intersubject correlation (ISC), visual perspective

## Abstract

**Introduction:**

Understanding how we perceive and interpret the movements of other living beings is fundamental to social interaction, survival, and navigating our dynamic environment. Traditional studies of biological motion perception have predominantly employed simplified stimuli, such as point-light displays, which sacrifice ecological validity for experimental control.

**Methods:**

To overcome this limitation, we created a novel stimulus set featuring naturalistic videos of isolated hand and leg movements. These stimuli incorporated variations in viewing perspective (first-person vs. third-person) and the presence or absence of object interactions, thereby increasing their real-world relevance. We recorded electroencephalography (EEG) from 28 adult participants as they viewed these goal-directed movements alongside nature scenes that served as control stimuli. Neural engagement was assessed through two complementary measures: inter-subject correlation (ISC), which captures shared neural responses across viewers, and intra-subject correlation (IaSC), which reflects temporal consistency of responses within individual viewers. We hypothesized that (1) biological limb movements would elicit stronger neural synchrony than non-biological motion, (2) movement type (arm vs. leg), object interaction, and visual perspective would differentially modulate ISC based on distinct affordance-related processing systems, and (3) these effects would replicate upon second exposure.

**Results:**

Our findings revealed that lower-limb movements elicited significantly greater ISC compared to both upper-limb movements and nature scenes. This effect proved robust, replicating consistently when participants viewed the same experimental conditions a second time. Furthermore, specific stimulus features modulated neural synchronization in domain-specific ways: first-person perspective enhanced ISC specifically for leg videos, whereas object interaction increased ISC specifically for arm videos. Notably, IaSC showed no differences across conditions, revealing a dissociation between cross-individual neural alignment and within-individual temporal consistency.

**Discussion:**

These findings provide new insights into how particular features of naturalistic movement systematically modulate shared neural engagement, advancing our understanding of biological motion perception in ecologically valid contexts. The dissociation between ISC and IaSC suggests that cross-individual neural synchronization and within-individual temporal consistency reflect distinct neural processes.

## Introduction

The perception of biological motion is a fundamental neurocognitive ability enabling social interaction and survival. Traditional research using point-light displays (Johansson, 1973) has identified a dedicated neural network for processing kinematic cues, centered on the superior temporal sulcus (Allison et al., 2000; Saygin, 2007). While these reductionist approaches have been instrumental in demonstrating that the visual system extracts sophisticated information from minimal motion cues, they provide limited insight into how the brain integrates the rich contextual features—such as environmental settings, object interactions, and spatial relationships—that characterize naturalistic human movements (Grosbras et al., 2012; Papeo et al., 2017). A central challenge is therefore to develop methodologies that balance experimental rigor with ecological validity (Hasson et al., 2004; Sonkusare et al., 2019).

We addressed this challenge using naturalistic video stimuli to examine action perception across development (Ntoumanis et al., 2021a, 2023b) and in clinical populations (Ntoumanis et al., 2022). Naturalistic stimuli offer superior ecological validity while maintaining precise neural measurement through inter-subject correlation (ISC; Hasson et al., 2004). However, a significant limitation of our prior work was incomplete stimulus control: videos varied substantially in content, duration, filming angle, and low-level visual features across categories (Ntoumanis et al., 2022). This uncontrolled variability introduced potential confounds, making it difficult to attribute observed ISC differences to specific, theoretically motivated movement parameters—such as limb type or object interaction—that are known to modulate action perception networks (Bachmann et al., 2018; Orban et al., 2021).”

The present study was designed to directly address this critical methodological limitation through the development of a rigorously controlled stimulus set aimed at systematically dissecting how specific movement features modulate neural synchrony in healthy adult observers, thereby illuminating the precise mechanisms underlying embodied perception. We created novel video stimuli that extend beyond simple movement-versus-static contrasts by systematically depicting distinct categories of upper-limb and lower-limb movements while carefully controlling for potential confounding variables. To ensure that any observed differences in neural synchronization could be attributed to the biological and functional properties of the movements themselves rather than to incidental differences in low-level visual properties (such as motion energy, luminance changes, contrast, or spatial frequency content) that can independently drive neural responses (Bartels and Zeki, 2004), we included a carefully matched category of nature scenes—featuring wind-driven movements of trees, grass, and water—as a dynamic control condition (Sonkusare et al., 2019). This nature category provides a critical baseline for isolating responses specifically related to biological motion perception as opposed to general visual motion processing. Critically, we maintained strict experimental control over filming conditions, including consistent camera angles, lighting, background settings, and video framing, to isolate and systematically manipulate specific action features. One such feature is the presence or absence of object interaction—a factor that fundamentally changes the semantic content and goal structure of an action and is known to modulate activity within action perception networks, particularly in regions involved in understanding action goals and tool use (Singh et al., 2025; Milani et al., 2025). By carefully varying whether the observed limb is interacting with an object or moving in isolation, we can examine how goal-directed object manipulation influences the neural processing of biological motion.

Visual perspective constitutes another critical and often underappreciated dimension of naturalistic action stimuli that has profound implications for how actions are processed and understood. Actions can be viewed from a first-person perspective (1PP), as if the observer is performing the action themselves and viewing their own body parts, or from a third-person perspective (3PP), as an external observer watching another person’s actions from outside their body. This distinction is far from trivial: first-person viewpoints typically elicit substantially stronger motor resonance—the automatic activation of the observer’s own motor system—and generate a greater subjective sense of embodiment and agency compared to third-person viewpoints (Vogt et al., 2003; Jackson et al., 2006; Gorisse et al., 2017). In other words, when we see an action from a first-person perspective, we tend to simulate it more strongly in our own motor system, almost as if we are preparing to execute it ourselves. These behavioral differences in motor resonance and embodiment are not merely subjective phenomena but reflect distinct underlying neural mechanisms: neuroimaging studies have consistently demonstrated that first- and third-person perspectives engage different patterns of activity in cortical regions responsible for body representation, self-other distinction, and motor planning (Ruby and Decety, 2001; Saxe et al., 2006). Given the fundamentally embodied nature of action perception—the idea that we understand others’ actions in part by simulating them in our own sensorimotor systems—systematically manipulating visual perspective alongside other stimulus dimensions such as limb type and object interaction allows us to examine how these factors jointly shape the neural synchronization that emerges during shared viewing experiences across multiple observers.

The concept of affordances—defined as the action opportunities that objects and environments offer to a perceiver—provides a valuable theoretical framework for understanding how perception of different body parts and their movements may differentially engage perceptual and motor systems. Research on “micro-affordances” has extensively documented how merely observing manipulable objects, even in the absence of any intention to act, automatically activates corresponding hand motor programs in a highly specific manner. For instance, viewing a cup with its handle oriented toward the right side facilitates subsequent right-hand grasping responses while inhibiting left-hand responses, reflecting the remarkably tight coupling between perceived object properties and the hand action representations that exist within reachable peripersonal space—the space immediately surrounding the body where manual interaction is possible (Tucker and Ellis, 1998). This automatic activation occurs rapidly and involuntarily, suggesting that hand-object affordances are processed as a fundamental aspect of visual perception. Complementing and extending this work, recent studies have introduced the concept of “macro-affordances” to describe how perception of large-scale navigable environmental layouts—such as open fields, corridors, or distant landscapes—preferentially activates locomotor programs and foot-related neural systems rather than hand-related systems. Behavioral and neurophysiological evidence has demonstrated that viewing panoramic vistas of distant, navigable space facilitates walking-related motor responses and modulates activity specifically in foot-related regions of dorsomedial motor cortex and parietal cortex involved in spatial navigation and postural control (Di Marco et al., 2019; Altomare et al., 2021; Tosoni et al., 2021). These complementary lines of research reveal a fundamental functional dissociation between limb-specific action systems that may have important implications for action perception: while hand actions are characterized by exceptional kinematic diversity and dexterity, enabling countless forms of fine-grained object manipulations ranging from threading a needle to playing a musical instrument, foot actions are primarily deployed for locomotion, postural support, balance maintenance, and gross spatial positioning through more stereotyped and biomechanically constrained motor patterns. This spatial reference frame distinction—with hands tightly coupled to peripersonal space and the manipulation of nearby objects, while legs are coupled to extrapersonal space and navigation through distant environments—suggests that upper- and lower-limb movements may engage fundamentally distinct neural processing systems. Consequently, these different limb types may elicit markedly different patterns of neural synchronization across observers during action perception, with potential differences in how reliably and uniformly different individuals process arm movements compared to leg movements.

Based on this theoretical background and empirical foundation, we formulated several specific hypotheses. First, we hypothesized that scenes depicting biological limb movements—whether of arms or legs—would elicit significantly higher ISC compared to control nature videos showing non-biological motion, reflecting the heightened attentional engagement, enhanced perceptual processing, and potentially stronger activation of mirror neuron systems that are triggered by biologically significant content relevant to understanding conspecifics. Second, we predicted that movement type (arm versus leg), the presence or absence of object interaction, and visual perspective (first-person versus third-person) would significantly modulate the degree of neural synchrony observed across viewers, with specific patterns revealing the selectivity and functional organization of the underlying neurocognitive mechanisms. For example, based on the affordance literature, we might expect that arm movements involving object interaction would show particularly high synchronization due to the automatic activation of hand-object motor programs, while leg movements might show different synchronization patterns reflecting their role in navigation and spatial positioning. Third, to rigorously assess the robustness and reliability of our findings, we employed a repeated-viewing paradigm in which participants viewed the same set of stimuli twice in succession, allowing us to test whether the observed patterns of neural synchronization were stable and replicable upon second exposure to the stimuli or whether they might change due to familiarity, habituation, or shifts in attention strategy.

This study thus aims to advance our fundamental understanding of biological motion perception by systematically examining how the brain’s response to controlled yet naturalistic actions is reflected in the inter-subject synchronization of neural activity measured through electroencephalography (EEG). By carefully manipulating theoretically motivated stimulus features while maintaining naturalistic viewing conditions, we seek to bridge the gap between highly controlled laboratory paradigms and real-world action perception (Smekal et al., 2024). Our methodological approach aligns with the growing emphasis in cognitive neuroscience on ecologically valid experimental paradigms that embrace principles of active vision, recognizing that oculomotor behavior, attention allocation, and neural processing are intrinsically linked and dynamically coordinated in the service of building coherent, actionable perceptual representations of the world (Nikolaev et al., 2025). By combining naturalistic stimuli with precise experimental control and quantitative measures of neural synchronization, our work offers a more nuanced and comprehensive view of how action perception operates under near-real-world conditions, moving the field closer to understanding how the brain actually processes the rich, complex movements we encounter in our daily lives.

## Methods

### Participants

Thirty healthy adults were recruited through social media advertisements to participate in the study. Data from two participants were excluded due to incomplete recordings caused by technical issues with stimulus presentation. The final sample consisted of 28 participants (15 females, aged 18–34 years, mean age = 23.4 years). All participants provided written informed consent prior to participation. The study was approved by the Institutional Review Board of the local ethics committee and conducted in accordance with the Declaration of Helsinki and its amendments.

### Stimuli

In Thirty-one video clips, each 24 s in duration, were used in this study. Twelve depicted upper-limb human movements (“arm” videos), twelve depicted lower-limb human movements (“leg” videos), and seven depicted natural scenes (“nature” videos). The arm videos comprised 6 with object interaction (3 from 1PP; 3 from 3PP) and 6 without objects (3 from 1PP; 3 from 3PP). Similarly, the leg videos comprised 6 with object interaction (3 from 1PP; 3 from 3PP) and 6 without objects (3 from 1PP; 3 from 3PP) (Supplementary materials S1–8).

The arm and leg videos were recorded by us for the purpose of this study. Filming was conducted in an indoor setting, using bright daylight lamps with diffusing filters to minimize harsh shadows and maintain uniform lighting. To standardize the temporal dynamics of the movements, the actor’s actions were paced with an audible metronome set to approximately 50 beats per minute (BPM). This audio served as a guide for the actor during recording only and was excluded from the final video stimuli; all clips were presented silently. This tempo was chosen to establish a deliberate and consistent rhythm, with minor adjustments for more complex actions to ensure the movements remained natural and fluid.

The actions were performed by two adolescent male actors. To maintain consistency within limb categories, a single actor performed all movements for a given limb type: upper-limb movements were performed by a 12-year-old, right-handed actor, and lower-limb movements were performed by an 11-year-old actor. As the actors were minors, written informed consent was obtained from their legal guardians, who were present throughout the entire recording session.

The activities shown included routine tasks, such as transferring beads into a container and wiping feet on a mat. Each frame was meticulously arranged to ensure that the active part of the limb was visible (at least from the elbow down for arms and the knee down for legs), and that all relevant objects and surfaces were fully in view. Extraneous elements within the frame were minimized to maintain focus on the target action.

Arm and leg videos were further categorized based on whether the movement involved interaction with an object, and on whether the camera angle was from 1PP or 3PP. In terms of object interaction, for the upper-limb (“arm”) videos, object interaction was defined as goal-directed manipulation of physical objects (e.g., transferring beads from a surface into a container) (Supplementary Figure 1B). Corresponding no-object control movements were kinematically matched but performed as pantomimes in empty space without physical objects (e.g., mimicking a precision pincer grasp and transfer motion in mid-air). This approach allowed us to isolate the effect of object presence while controlling for movement kinematics. For the lower-limb (“leg”) videos, object interaction was defined as direct physical contact with a discrete object or surface that constrained or guided the movement (e.g., wiping a foot on a doormat), in contrast to unconstrained movements performed in empty space (e.g., stepping motions without surface contact).

For the 1PP condition, the camera was mounted to capture a first-person egocentric perspective, with the camera positioned to simulate the viewpoint of an actor looking down at their own limbs during action execution. This egocentric viewpoint aimed to maximize embodiment and motor resonance by matching the natural visual experience during self-generated actions. For the 3PP condition, the camera was positioned at a fixed location, approximately 1.5 meters away from the actor at a 45-degree angle, where limbs were filmed from an external observer’s viewpoint, providing a more allocentric spatial representation of the movement. This manipulation allowed us to test whether perspective interacts with limb type and object interaction to modulate neural synchrony.

The nature videos were selected from openly licensed stock footage^1^ to be comparable in visual complexity to the action videos. We specifically chose clips with slow, continuous camera motion (e.g., gentle pans across a landscape) and avoided scenes containing salient, discrete events, humans, or animals. The content primarily consisted of landscapes, such as fields, clouds, and flowing water. A complete list and description of all stimuli are provided in Supplementary Table 1. Importantly, these nature scenes depicted panoramic landscapes with slow camera motion providing visual information that contains implicit macro-affordances for spatial navigation (Di Marco et al., 2019) while lacking explicit biological motion. This design allowed us to examine whether environmental spatial properties alone in the absence of human movement can drive neural synchronization comparable to observing limb actions.

### Procedure

The experiment consisted of six blocks, each lasting 6 min. In each block, participants viewed 15 video clips (24 s each) presented without inter-trial intervals. A short break of 30–90 s was given between blocks. The last three blocks contained the same set of videos as the first three blocks, allowing us to test the replicability of our findings. Data corresponding to the first three blocks are referred to as the “first viewing” and data from the last three blocks as the “second viewing” (Supplementary Figure 1A).

The order of blocks within each viewing was randomized across participants, but the order of video clips within each block was fixed to maintain a controlled presentation order across conditions. Specifically, each block began with either an arm or leg movement video, followed by a nature video, then a movement video of the opposite type, and so on, ensuring that a nature video always separated movement videos to reduce potential carry-over effects. This interleaving ensured that non-biological dynamic scenes, which lack the affordance-related motor activation elicited by observed limb movements, served as a perceptual baseline between successive biological motion clips.

All videos were presented without audio, as participants simultaneously performed a non-attended auditory oddball task, the results of which will be separately reported elsewhere. In this task, participants heard a sequence of spoken words, some of which had semantic associations with arm or leg movement, while others were unrelated or pseudowords. Because of this semantic overlap, we conducted a control analysis to verify that the frequency of each oddball category (arm-related, leg-related, neutral/pseudowords) was similar across the video categories (arm, leg, nature). This analysis confirmed that oddball categories were evenly distributed across video conditions (Supplementary Table 2), indicating that the oddball task was unlikely to have systematically influenced the EEG results reported here. Overall, the experiment lasted approximately 40 min, and all participants received monetary compensation for their time.

### EEG data collection and preprocessing

EEG activity was recorded continuously using a BioSemi ActiveTwo system at a sampling frequency of 500 Hz. Participants were fitted with a standard 32-electrode cap following the international 10–20 system, with Cz as the reference electrode. To subsequently remove eye-movement artifacts, the vertical and horizontal electrooculogram (VEOG and HEOG) were also recorded with two auxiliary electrodes (one placed below one eye and the other placed laterally to the opposite eye; Ntoumanis et al., 2023a). Precise temporal alignment between stimulus presentation and EEG recording was achieved by having the stimulus presentation software [PsychoPy; Peirce et al., 2019)] send triggers via a parallel port at the onset of each video clip. These triggers were synchronized with the screen refresh rate to capture actual rather than nominal stimulus onsets.

The EEG data were preprocessed following a pipeline established in recent studies (Cohen and Parra, 2016; Ntoumanis et al., 2021a, 2023a, 2023b). First, the data were re-referenced to the average reference (Ntoumanis et al., 2023a), downsampled at 250 Hz, notch-filtered at 50 Hz, and band-pass filtered between 1 and 50 Hz (Madsen and Parra, 2022; Ntoumanis et al., 2023a). Noisy channels (mean = 2.7 per recording) were detected by visual inspection and interpolated from surrounding electrodes (Ki et al., 2016; Ntoumanis et al., 2023a). Eye-movement artifacts were removed using Independent Component Analysis (ICA) with the infomax algorithm (Bell and Sejnowski, 1995). Outlier samples exceeding 4 SDs of the mean of their respective channel were replaced with 0, along with a 40 ms window before and after each outlier (Cohen and Parra, 2016; Ntoumanis et al., 2023b).

All preprocessing was performed in MNE Python (v1.0.3; Gramfort, 2013). Interactive HTML reports were generated for each recording, containing detailed documentation of the preprocessing steps. These reports are available at https://osf.io/4x98u/files/osfstorage.

### Intersubject correlation analysis

EEG inter-subject correlation was measured using correlated component analysis (CCA; Parra et al., 2019), a multivariate technique that identifies linear combinations of EEG channels that maximize correlation across participants. Preprocessed EEG segments corresponding to individual video clips were extracted based on trigger markers embedded in the continuous EEG recording and then concatenated across participants to form multi-subject data matrices. For each video clip, between-subject and within-subject covariance matrices were computed from the concatenated data. These covariance matrices were then averaged across all videos within each condition, yielding a single, representative set of projection vectors that could be applied uniformly to all stimuli (Cohen and Parra, 2016). This averaging approach enhances the stability and generalizability of the ISC estimation by ensuring that the derived components capture consistent patterns of synchronization across multiple exemplars rather than idiosyncratic features of individual clips (Nastase et al., 2019).

Based on these average covariance matrices, the correlated components—the spatial filters that maximize between-subject correlation relative to within-subject variability—were optimized using generalized eigenvalue decomposition. The ISC for each participant was then computed using a leave-one-out cross-validation approach, in which each participant’s neural activity was correlated with the average activity of all other participants, excluding that individual. This procedure prevents circularity and provides an unbiased estimate of how well each participant’s brain activity aligns with the group response. The leave-one-out approach resulted in a single ISC value per participant per condition, reflecting the degree to which that participant’s neural activity was synchronized with the collective neural response of the group.

The reported ISC values correspond to the sum of the three most correlated components, following established practices in previous ISC studies (Iotzov et al., 2017; Cohen et al., 2018; Petroni et al., 2018). This composite measure allows us to capture the overall level of neural synchronization across multiple components without requiring assumptions about which specific component is most relevant, and it is robust to potential differences in the anatomical origin or functional significance of individual components across conditions. The short clip length of 24 s per video was sufficient for reliable ISC estimation, consistent with prior methodological work demonstrating that ISC can be robustly measured from naturalistic stimuli of similar or even shorter durations (Madsen and Parra, 2022).

For the purposes of visualizing and interpreting the spatial distribution of neural synchronization, we also computed projection vectors (spatial filters) and forward models separately for each experimental condition (Cohen and Parra, 2016). While projection vectors describe how EEG sensor data are linearly combined to form correlated components, forward models describe the inverse relationship – specifically, how each component couples to or projects onto the EEG sensors. Forward models provide more neurophysiologically interpretable maps of the spatial distribution of each correlated component’s activity on the scalp, as they are less affected by volume conduction and better reflect the underlying source configuration. By computing these forward models separately for each condition, we were able to visualize and systematically compare the scalp topographies of the three strongest correlated components across the arm, leg, and nature video categories, potentially revealing condition-specific patterns of neural engagement.

### Average luminance difference calculation

When comparing ISC across video categories, differences in low-level visual dynamics could act as a confounding factor (Ntoumanis et al., 2023b). In this study, given that the arm and leg movement videos were recorded in a controlled environment with consistent light, these videos might naturally contain less frame-to-frame changes in luminance than videos depicting natural scenes. To control for this, we quantified the overall visual dynamics of each video using the average luminance difference (ALD; Poulsen et al., 2017; Ntoumanis et al., 2023a). Specifically, for each video, we extracted all frames and converted them to grayscale (0–255) by averaging over the RGB channels. We then computed the absolute luminance difference between each frame and the preceding frame, averaged these differences across all pixels, and finally averaged across the entire video to obtain a single ALD value. Higher ALD values thus reflect greater overall temporal change in luminance. The ALD was computed in Python using the OpenCV library for video processing and frame conversion (von Kleist-Retzow et al., 2016). This metric was then used as a covariate in subsequent statistical analyses to control for the effect of visual dynamics when examining differences in ISC between video categories, ensuring that any observed differences in neural synchronization could be attributed to the biological or semantic properties of the stimuli rather than to incidental differences in low-level visual motion or luminance change.

## Statistical analysis

First, we examined whether there was a statistically significant association between ISC and ALS, as reported in previous studies (Poulsen et al., 2017; Ntoumanis et al., 2023b). Following a significant correlation, we regressed ISC values on ALD and used the resulting residuals as ALD-controlled ISC measures for all subsequent analyses. Using these residuals, a one-way repeated measure analysis of variance (ANOVA) was conducted to compare the ISC between the three video categories (arm, leg, and nature). Throughout the manuscript, we report classic eta-squared (*η*^2^) effect sizes for ANOVA results, rather than partial or generalized *η*^2^, in accordance with standard practice for the analyses conducted. Post-hoc analysis included paired-samples *t*-tests to examine whether the ISC was statistically different between all pairs of conditions. The false discovery rate (Benjamini and Hochberg, 1995) was applied to control for multiple comparisons and reduce the likelihood of Type I errors. This analysis was conducted separately for the first and the second viewing to rigorously test the replicability and stability of our findings across repeated exposures to the same stimuli. The same analytical approach was also performed to compare the intra-subject correlation (IaSC) between conditions, allowing us to assess whether within-individual temporal consistency of neural responses showed similar or different patterns compared to the across-individual synchronization measured by ISC.

Moreover, we estimated linear regression models to examine the effects of point-of-view (POV; first-person versus third-person perspective) and object interaction (present versus absent) on ISC. Since only the arm and leg videos contained systematic variation in these parameters, data from the nature videos were not included in these regression analyses. Several candidate models were estimated, each incorporating different combinations of main effects and interaction terms (e.g., limb type × POV, limb type × object interaction, POV × object interaction, and the three-way interaction limb type × POV × object interaction), and these models were compared to one another based on Akaike’s Information Criterion (AIC). The AIC provides a principled approach to model selection by balancing goodness of fit with model complexity, with lower AIC values indicating better models. This model comparison approach allowed us to identify which combinations of factors and their interactions best explained the observed patterns of neural synchronization (Supplementary Figure 1B).

## Results

**Figure 1:**
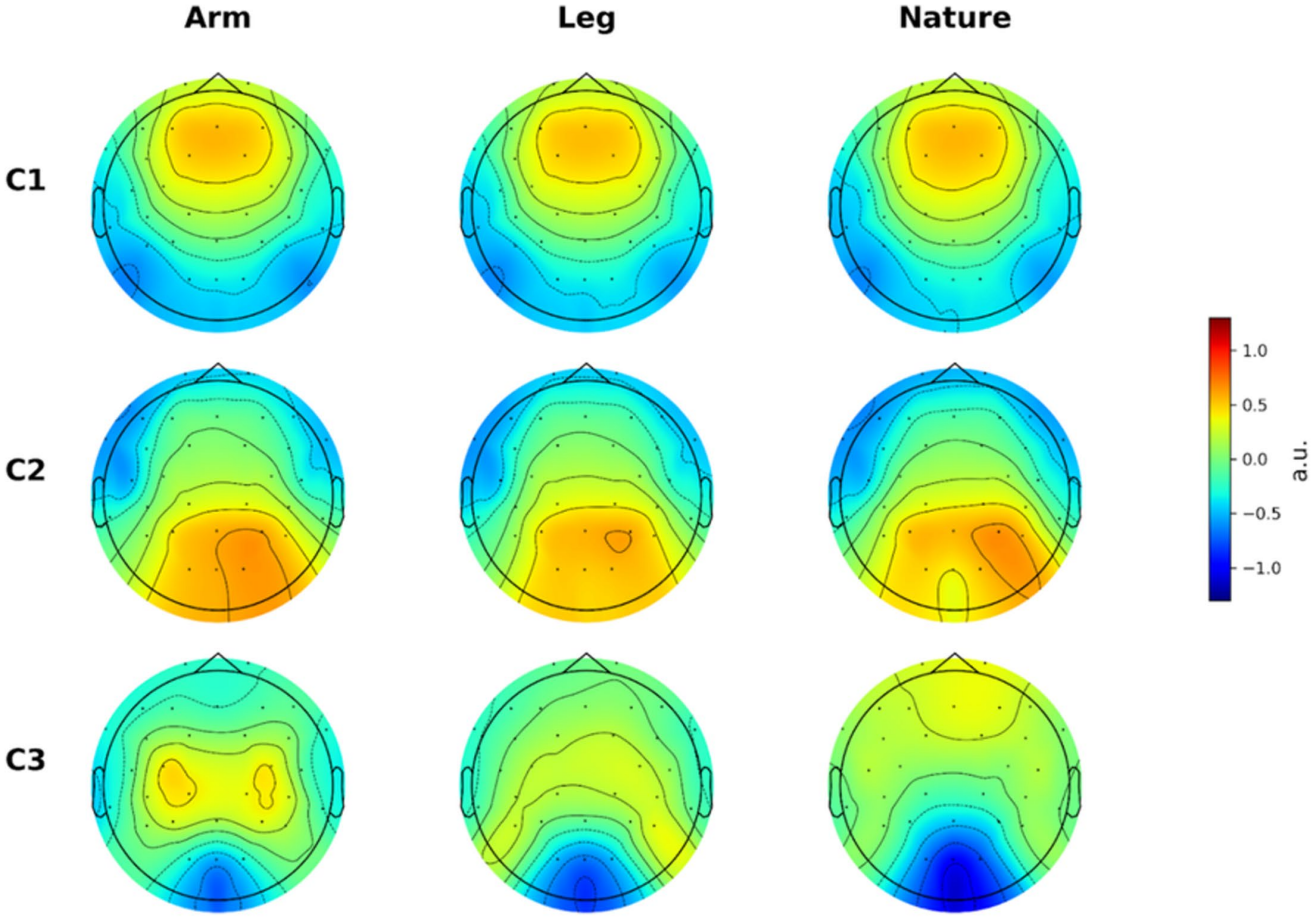
Inter-subject correlation component topographies for naturalistic action perception.

First, we analyzed data from the first viewing only, that is, data corresponding to the video’s participants watched for the first time during the experiment. We considered this to reduce the effect of prior exposure to the videos on the resulting ISC (Dmochowski et al., 2012; Chang et al., 2016). We estimated the components of the EEG signals that capture the highest ISC for each condition separately (Figure 1).

Interestingly, the overall pattern of scalp topographies was qualitatively similar across conditions, suggesting that the neural mechanisms giving rise to ISC were broadly analogous among the arm, leg, and nature video categories. Specifically, the first component (C1) demonstrated a predominantly frontal topography, possibly reflecting the engagement of higher-level attentional, executive, or semantic processes that are time-locked across viewers as they interpret and make sense of the observed actions. The prominence of frontal areas in C1, rather than the visual areas that typically dominate ISC in more passive viewing paradigms, may reflect the goal-directed, motor-perceptual nature of our task, where participants actively processed action-relevant information rather than simply registering visual features. The second component (C2) showed a clear occipital positivity, likely reflecting synchronized low-level visual processing or early sensory responses to the stimulus features. The ordering of C1 and C2—with higher-order frontal processes showing stronger synchronization than lower-level sensory responses—could be explained by the goal-directed attention and action understanding that our task emphasized, making higher-order cognitive processes more consistently time-locked across participants than the more variable lower-level sensory responses that might depend on individual differences in visual attention or eye movements. Finally, the third component (C3), where the greatest differences among conditions were observed, showed an occipital negativity and, particularly pronounced in the arm condition, a parietal positivity in regions close to sensorimotor cortex. Overall, the scalp projections of the correlated neural activity suggest that perceptual areas (primarily occipital cortex), motor-related areas (parietal and central regions), and higher-order cognitive areas (frontal regions) all contributed substantially to the inter-subject correlation, consistent with a distributed network supporting naturalistic action perception.

**Figure 2:**
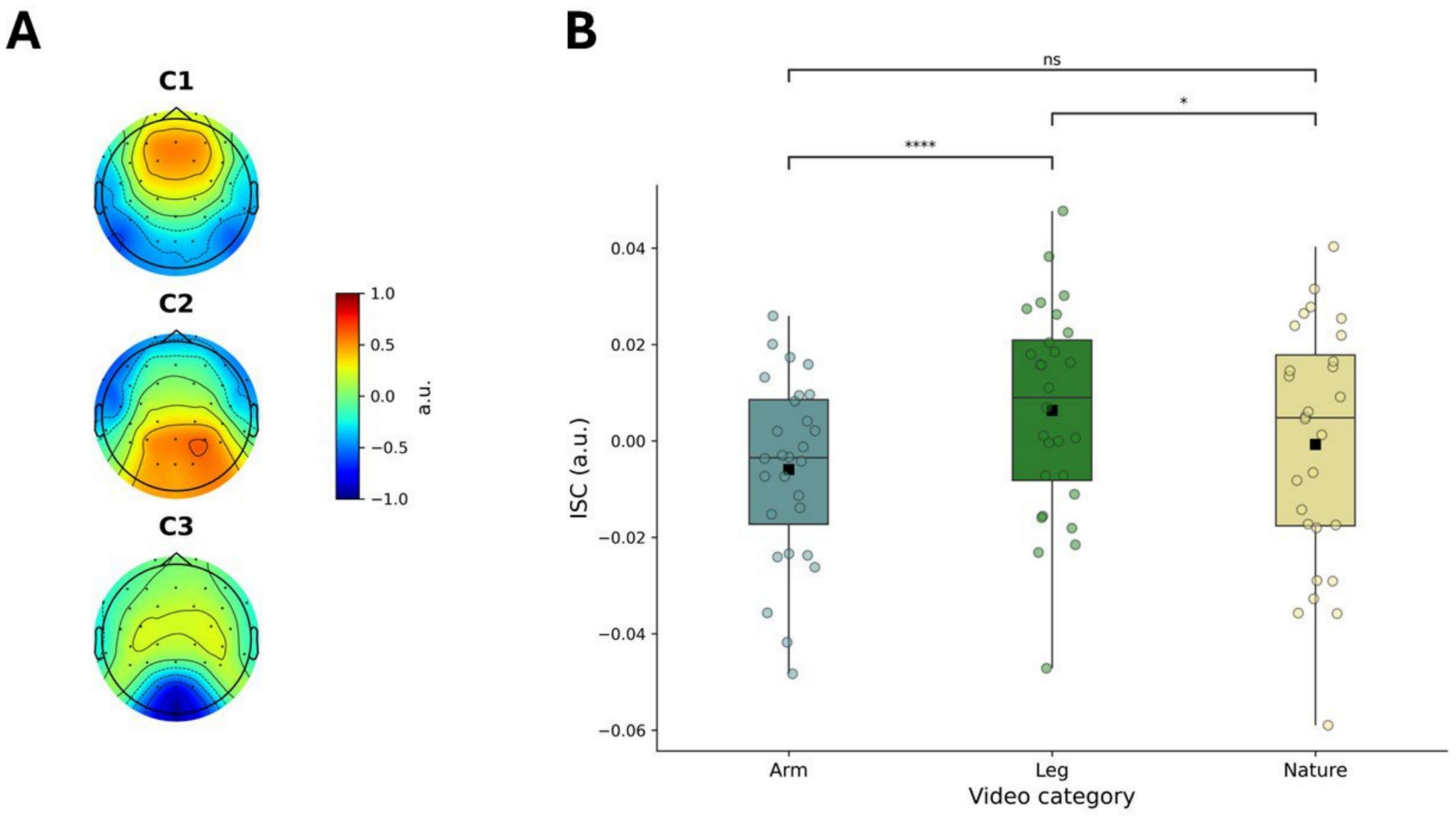
Condition-specific inter-subject correlation after controlling for alpha lateralization: **(A)** Spatial filters for inter-subject correlation derived from the pooled data across all conditions; **(B)** Condition-specific ISC values after controlling for average luminance difference (ALD). *p < 0.05, ***p < 0.0001.

Next, we sought to examine whether different conditions elicited different levels of ISC. For that, the same set of projection vectors had to be estimated by pooling data from all conditions (Figure 2A; Cohen and Parra, 2016). To account for potential confounding by ALD, we first calculated the correlation between ISC and ALD, which was found to be statistically significant (*r* = 0.278, *p* = 0.033). Therefore, we first regressed ISC values on ALD and used the resulting residuals as ALD-controlled ISC measures. After controlling for ALD, the mean ISC residuals were highest for leg videos (M = 0.005, SD = 0.06, CI [−0.018, 0.028]), followed by arm videos (M = 0.001, SD = 0.06, CI [−0.022, 0.024]), and were lowest for nature videos (M = −0.009, SD = 0.05, CI [−0.03, 0.012]). Using these residuals, a one-way repeated measures ANOVA revealed a significant effect of Condition on ISC (*F*(2,54) = 11.596, *p* < 0.0001, *η*^2^ = 0.052, CI [0.018, 0.087]), after controlling for ALD (Figure 2B). Post-hoc analysis showed that leg videos evoked greater ISC than arm videos (*t* = 7.818, p < 0.0001, *d* = 0.607, CI [0.417, 0.789]) and nature videos (*t* = 2.241, *p* = 0.033, *d* = 0.302, CI [0.068, 0.531]). Finally, nature videos elicited greater ISC than arm videos, although this difference did not reach statistical significance (*t* = 1.937, *p* = 0.063, *d* = 0.236, CI [0.027, 0.441]).

Moreover, a linear regression was estimated to test the effect of POV and object interaction on ISC. Since only the arm and leg videos had these parameters, data from the nature videos were not included in this analysis. Several models were estimated, each time including different interaction terms, and compared to one another based on Akakie’s Information Criterion (AIC). Table 1 summarizes the results of the model that best fitted the data (lowest AIC). In line with the ANOVA results, the regression showed higher ISC during watching leg videos compared to arm videos (*p* = 0.008). In addition, arm videos featuring an interaction with an object elicited greater ISC than arm videos without object interaction (*p* = 0.004). However, this effect was significantly less significant in the leg videos (*p* = 0.016). Conversely, a 1PP camera angle increased the ISC while watching leg videos (*p* = 0.006), but not while watching arm videos (*p* = 0.221). These findings demonstrate that the factors enhancing neural synchronization differ systematically between upper- and lower-limb action perception, with object interaction being critical for arm movements and egocentric perspective being critical for leg movements.

**Table 1:** Linear regression model predicting inter-subject correlation during upper- and lower-limb action perception.

Effect	Estimate	SE	95% CI		*p*
			LL	UL	
ALD	0.005	0.004	−0.002	0.012	0.199
Leg	0.012	0.005	0.003	0.021	0.008**
Object	0.011	0.004	0.004	0.018	0.004**
POV	0.007	0.006	−0.004	0.018	0.221
Leg × object	−0.013	0.005	−0.024	−0.003	0.016*
Leg × POV	0.018	0.007	0.005	0.031	0.006**

The model shows the effects of average luminance difference (ALD), action type (leg vs. arm), object interaction (presence vs. absence), and point-of-view (POV; 1st-person vs. 3rd-person) on ISC. CI = confidence interval; LL = lower limit; UL = upper limit. *p < 0.05, **p < 0.01.

**Figure 3:**
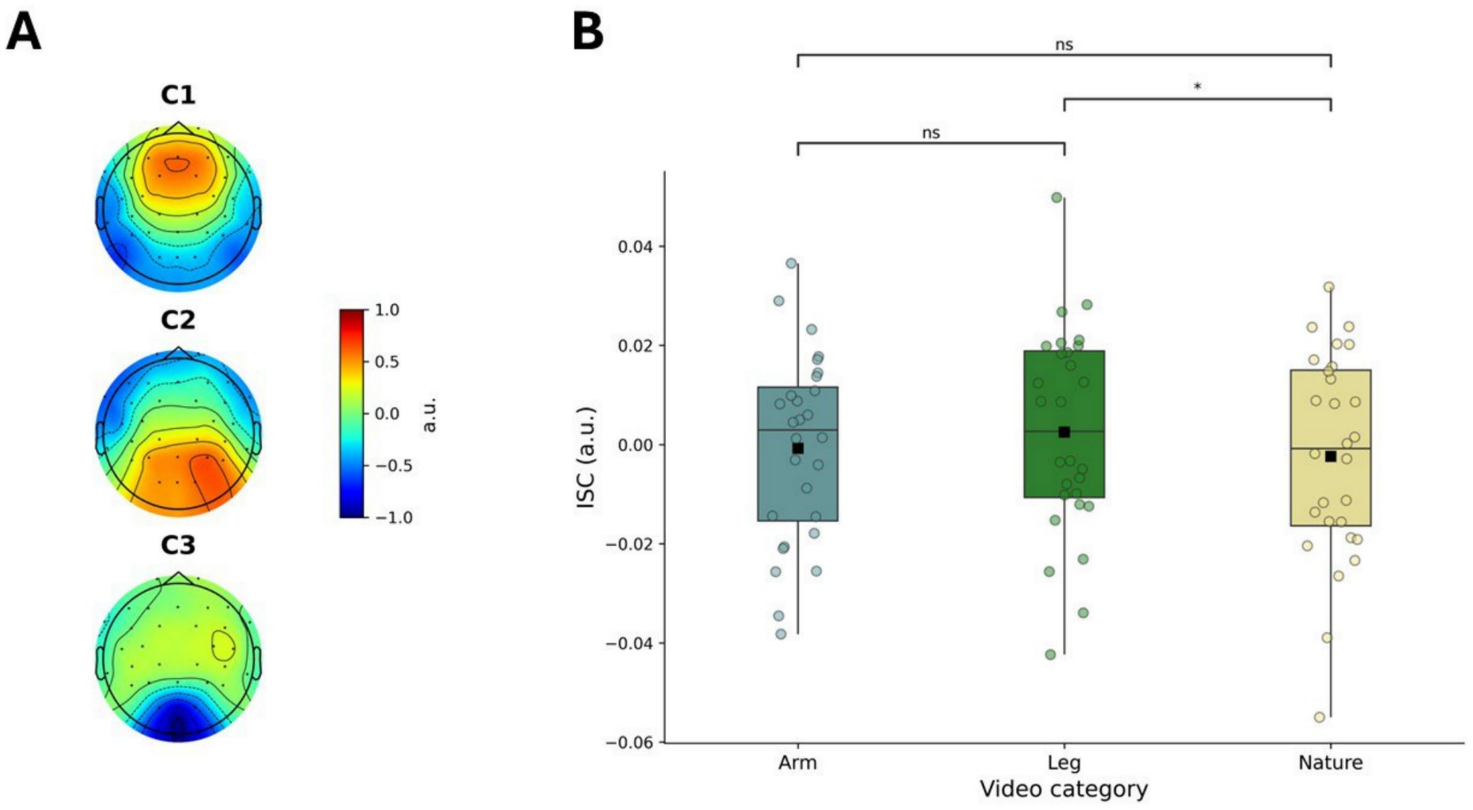
Replicability of neural synchronization during second video viewing. **(A)** Scalp topographies of the inter-subject correlation (ISC) components from the second viewing, demonstrating a pattern similar to the first viewing; **(B)** Condition-specific ISC values from the second viewing after controlling for average luminance difference (ALD). *p < 0.05.

Given that our experimental design enabled participants to watch the videos multiple times, we used data from the second viewing to test the replicability of the results obtained from the first viewing. The scalp topographies were similar to the ones estimated from the first viewing (Figure 3A), thus suggesting that the same electrode sites contributed the most to the ISC, irrespective of the viewing. A one-way repeated measures ANOVA revealed a significant effect of Condition on ISC (*F*(2,54) = 3.928, *p* = 0.026, *η*^2^ = 0.010, CI [0.001, 0.024]), after controlling for ALD (Figure 3B). Post-hoc analysis showed that leg videos evoked greater ISC than nature videos (*t* = 2.526, *p* = 0.018, *d* = 0.233, CI [0.071, 0.391]), consistently with the first viewing. In addition, there was a trend towards significance when comparing the ISC between leg and arm videos (*t* = 1.912, *p* = 0.067, *d* = 0.161, CI [0.016, 0.303]) in the same direction as found in the first viewing. Lastly, the ISC was not significantly different between arm and nature videos (*t* = 0.978, *p* = 0.337, *d* = 0.082, CI [−0.058, 0.220]), in line with the result from the first viewing.

Further analysis compared the ISC between the first and the second viewing. When data from all conditions were combined, the ISC was not significantly different between the two viewings (*t* = 0.082, *p* = 0.935, *d* = 0.005, CI [−0.095, 0.105]). However, when considering each condition separately, we found that the ISC while watching arm videos increased in the second viewing relative to the first one (*t* = 3.230, *p* = 0.003, *d* = 0.274, CI [0.119, 0.424]). This effect was not observed for the leg videos (*t* = 1.928, *p* = 0.065, *d* = 0.181, CI [0.020, 0.339]) or the nature videos (*t* = 0.536, *p* = 0.596, *d* = 0.072, CI [−0.150, 0.293]).

**Figure 4:**
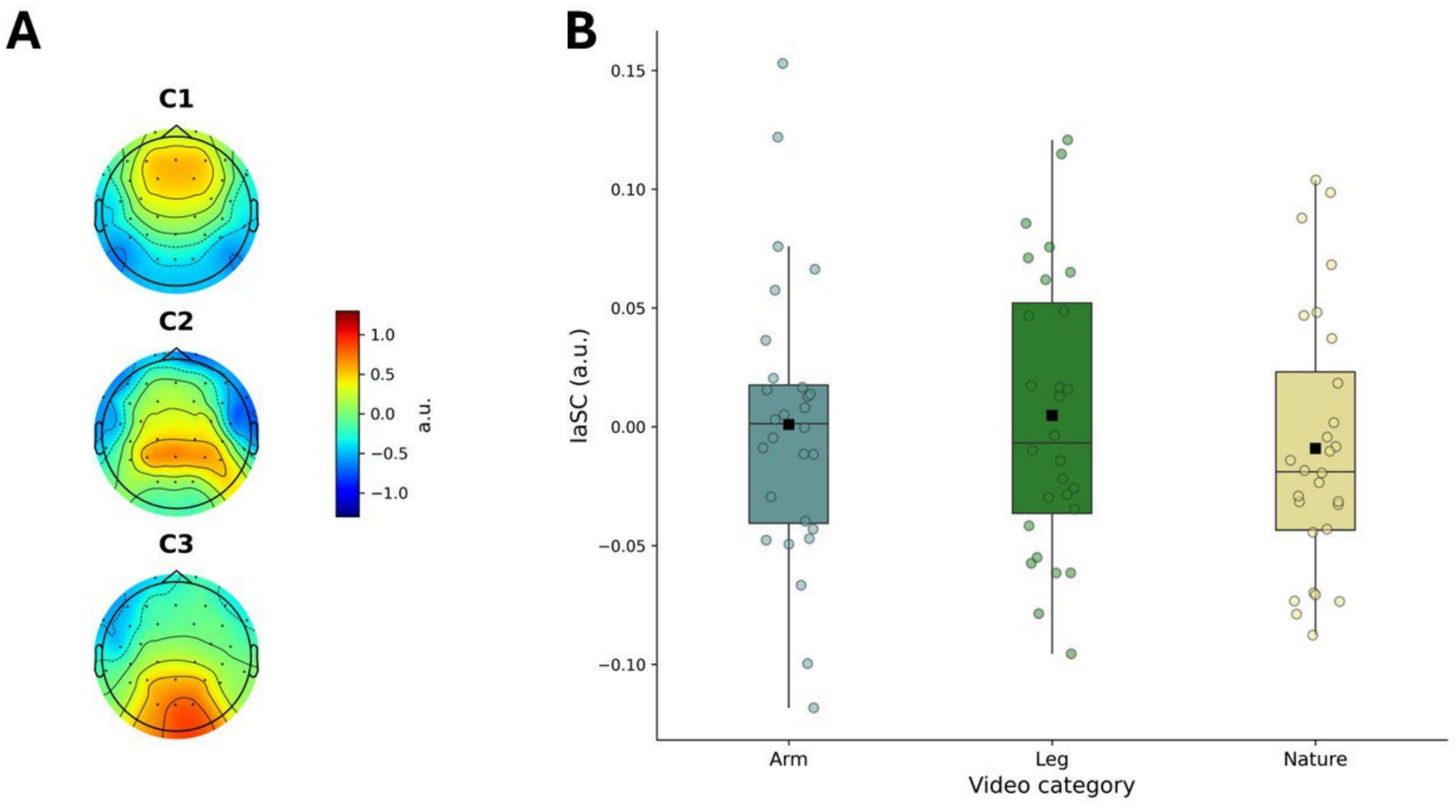
Intra-subject correlation (IaSC) and cross-viewing reliability. **(A)** Scalp topographies of the components maximizing neural correlation within subjects across viewings (IaSC); **(B)** Condition-specific IaSC values after controlling for average luminance difference (ALD).

Finally, we sought to examine whether different conditions elicited different levels of IaSC. For this analysis, the correlated components were estimated separately to maximize the neural synchrony within subjects and across viewings. The scalp topographies were very similar to the ones obtained when maximizing the ISC (Figure 4A), suggesting that the neural sources responsible for the correlated activity across subjects leads to within-subject reliability. However, a one-way repeated measures ANOVA showed no significant effect of Condition on IaSC (F(2,54) = 1.108, *p* = 0.337, *η*^2^ = 0.011, CI [0.000, 0.040]), after controlling for ALD (Figure 4B).

To address concern regarding the temporal standardization of the movement stimuli and its potential influence on ISC, we performed an additional analysis of the rhythmic structure of the videos and its relationship to neural synchrony.

For each video, optical flow was computed frame-by-frame to estimate motion vectors. The magnitude of the optical flow field was averaged across pixels to obtain a motion-energy time series. We then computed the power spectrum of this time series and defined a Rhythmicity Index (RI) as the normalized spectral power at the target pacing frequency (0.5–1.5 Hz; corresponding to approximately 50 BPM). Thus, RI captures the strength of periodic modulation in stimulus motion at the metronome frequency.

Analysis of RI values across conditions revealed distinct rhythmic profiles. The Arm condition exhibited a pronounced peak at approximately 0.8 Hz, confirming successful temporal entrainment by the metronome. The Leg condition showed a broader distribution of rhythmicity, though RI values remained clearly elevated relative to the Nature condition, which contained no biological movement and minimal periodic structure. The broader distribution in the Leg condition likely reflects biomechanical and kinematic differences inherent to lower-limb movements—specifically, greater mass and inertia, as well as more complex acceleration–deceleration dynamics—which introduce variability in motion-energy periodicity despite external pacing. Importantly, both movement conditions demonstrated higher rhythmicity than the Nature condition, confirming effective temporal standardization of the stimuli.

To test whether ISC was directly driven by rhythmic strength, we examined the correlation between ISC and RI across all stimuli. This analysis yielded a non-significant correlation (*r* = −0.25, *p* = 0.178), indicating that the observed ISC effects are unlikely to reflect low-level stimulus periodicity. Notably, prior to ISC computation, EEG signals were bandpass filtered between 1 and 50 Hz, which likely contributed to attenuation of the rhythmic component.

## Discussion

The present study employed inter-subject correlation (ISC) analysis of EEG to investigate the neural dynamics underlying the observation of naturalistic human motor actions. We systematically examined how movement type (upper- versus lower-limb), contextual features (point-of-view perspective, object interaction), and low-level visual properties modulate neural synchrony across viewers. Our findings reveal a complex pattern of condition-specific synchrony, robust replicability across viewings, and a fundamental dissociation between inter-subject (ISC) and intra-subject (IaSC) correlation measures.

Consistent with prior research using naturalistic stimuli, observation of all video categories elicited significant and reliable ISC in EEG signals (Dmochowski et al., 2012; Ki et al., 2016). This indicates that the temporal dynamics of neural responses were substantially synchronized across participants, suggesting a shared mechanism of perceptual and cognitive processing regardless of stimulus content (Hasson et al., 2004). Critically, the strength of this neural synchronization was significantly modulated by video category. After statistically controlling for low-level visual features using the Average Luminance Difference (ALD) metric, videos depicting lower-limb (leg) movements evoked significantly stronger ISC than both upper-limb (arm) movement videos and nature videos. No statistically significant difference in ISC was found between arm movement videos and nature videos. This finding can be interpreted through the concept of “macro-affordances,” which refers to the automatic facilitation of locomotion-related actions upon perceiving distant spatial layouts (Di Marco et al., 2019; Tosoni et al., 2021). Leg movements, inherently linked to whole-body navigation within extrapersonal space, likely elicit more consistent and shared neural responses across observers by activating a universal mechanism for processing navigation-relevant cues. Conversely, upper-limb movements, associated with object manipulation within peripersonal space, tend to invoke more variable, context-dependent interpretations, resulting in lower neural synchronization (Di Marco et al., 2019). Research demonstrates that walking behavior enhances peripheral visual processing by reducing alpha oscillations, thereby diminishing inhibitory control and expanding attentional focus crucial for navigation (Cao and Händel, 2019).

Given that leg movements are closely tied to this functional brain state, stimuli depicting such actions are more likely to engage a universal neural network across individuals, whereas arm movements involve more individualized cognitive processing, leading to decreased inter-subject neural synchrony.

The finding that first-person perspective (1PP) specifically enhanced ISC for leg videos (*p* = 0.006) but not arm videos (*p* = 0.221) reveals a limb-specific, viewpoint-dependent mechanism that we attribute to the interaction between ecological viewing experience and action novelty. First, individuals observe their own feet predominantly from a first-person, downward-looking viewpoint during everyday activities (e.g., walking, sitting, navigating stairs, or tying shoes). This creates extensive perceptual-motor experience with feet viewed from an egocentric frame of reference. When participants in our study observed foot actions from 1PP, this viewpoint matched their habitual visual experience of monitoring their own feet, potentially facilitating more automatic and uniform embodied simulation across individuals—activating foot motor representations “as if” performing the action themselves (Ruby and Decety, 2001; Jackson et al., 2006). Critically, this familiar egocentric viewpoint was applied to highly unusual manipulative actions: participants watched feet grasping objects, rolling balls, and wiping floors with toes—motor behaviors rarely performed or observed in daily life. The combination of familiar viewing perspective (1PP) with novel motor content created a synergistic effect that may have maximized attentional engagement uniformly across observers, thereby elevating ISC. This novelty-attention hypothesis is central to understanding our results. Foot manipulations are inherently more salient and attention-capturing than hand manipulations: while observing someone using their hands to grasp, move, or manipulate objects is ubiquitous in everyday visual experience, observing feet performing the same actions is unusual and surprising. This differential novelty may drive more consistent, stimulus-driven attentional allocation for foot actions compared to hand actions, reducing individual variability in where and when attention is deployed during viewing. The 1PP viewpoint amplifies this effect for legs by providing an embodied, egocentric frame that enhances the sense of “I am doing this unusual action,” whereas 3PP introduces spatial distance (“someone else is doing this unusual action”) that may weaken embodied engagement and increase processing variability across individuals.

Importantly, this viewpoint sensitivity was absent for hand actions (*p* = 0.221). We attribute this null effect to two factors. First, hands are extensively observed from both first-person (during one’s own manual tasks) and third-person (during social interactions, demonstrations, collaborative work) perspectives in daily life, creating balanced visual-motor experience across viewpoints. This may render participants equally proficient at embodied simulation of hand actions regardless of perspective. Second, hand manipulations in our videos (e.g., grasping, moving, transferring objects) were familiar and routinely observed actions, lacking the attentional novelty of foot manipulations. Without the novelty-driven attentional capture that characterized foot videos, the perspective manipulation had minimal impact on ISC for arm videos. Thus, the 1PP × limb interaction demonstrates that perspective-dependent embodiment is not a universal property of biological motion perception, but rather emerges from the interplay between (1) ecological viewing statistics (which viewpoints are typically experienced for a given body part) and (2) action novelty (how unusual the observed behavior is). When a familiar viewpoint (1PP for feet) is paired with novel behavior (foot manipulations), maximal neural synchronization is achieved through enhanced embodied attention.

The intermediate ISC values for nature videos—significantly lower than leg videos (*p* = 0.033) yet showing a numerical trend toward higher values than arm videos (*p* = 0.063)—provide insight into the factors driving neural synchronization. Nature videos in our study depicted ecologically universal visual content: panoramic landscapes, flowing water, moving clouds, and slow camera pans across natural scenes. Such stimuli possess several properties that may promote relatively uniform neural responses across observers. First, natural scenes engage low-level visual processing mechanisms (motion detection, spatial frequency analysis, depth perception) that are highly conserved across individuals and driven largely by bottom-up stimulus properties rather than top-down interpretative processes (Bartels and Zeki, 2004; Hasson et al., 2004). The continuous optical flow generated by camera motion and environmental dynamics (water flow, cloud movement) may elicit consistent activation of motion-sensitive visual areas (MT+/V5) across viewers, contributing to moderate ISC. Second, panoramic landscape views may engage similar neural mechanisms to those involved in processing spatial distance for navigation. Research has demonstrated that perception of distant (versus near) targets in environmental layouts facilitates walking-related locomotion and activates sensory-motor systems coding spatial distance (Di Marco et al., 2019; Tosoni et al., 2021). While these studies used controlled environmental layouts rather than natural scenes, the presence of distant horizons and traversable terrain in nature videos may engage related neural systems involved in coding navigable space and planning potential locomotor trajectories. Third, natural scenes are inherently ecologically familiar yet perpetually variable: while humans have extensive experience viewing natural environments (evolutionarily relevant visual content), each specific landscape provides novel configurational details that sustain attention without the interpretive ambiguity of abstract stimuli. This balance between familiarity and novelty may promote moderate, sustained engagement across viewers.

However, the critical observation is that nature videos, despite these properties, elicited significantly lower ISC than leg videos (*p* = 0.033). This dissociation reveals that explicit biological motion, particularly of effectors associated with locomotion and unusual manipulations, drives stronger neural synchronization than environmental affordances or low-level visual dynamics alone. The trend for nature videos to exceed arm videos (*p* = 0.063), though not statistically significant, is consistent with the hypothesis that spatial affordances and low-level visual consistency can partially compensate for the absence of biological motion. Conversely, arm videos, despite containing explicit biological motion and motor resonance, may suffer from high interpretive variability due to the enormous kinematic diversity of hand actions and their familiar, low-salience nature. This pattern suggests that ISC magnitude depends on the balance between stimulus-driven attentional capture, motor resonance, and interpretive constraint.

The finding that object interaction significantly enhanced ISC for arm videos aligns with the notion that the presence of a tangible goal provides a more constrained and unambiguous kinematic pattern, reducing interpretative variability across observers and leading to more shared neural representations (Di Marco et al., 2019; Casartelli et al., 2023). When observing hand-object interactions, the physical constraints imposed by object properties (shape, size, weight) and task demands (precision requirements, spatial targets) narrow the space of possible action interpretations, promoting more uniform cognitive processing across individuals. This is consistent with the micro-affordance literature demonstrating that manipulable objects automatically activate specific hand motor programs (Tucker and Ellis, 1998). In contrast, pantomimed arm movements without objects may be interpreted more variably—as communicative gestures, abstract expressions, or ambiguous motor patterns—thereby reducing neural synchronization.

Conversely, the attenuated effect of object interaction for leg videos suggests that the presence of physical objects or surfaces does not equivalently constrain the interpretation of lower-limb actions. This may reflect the fact that leg movements, even without objects, are strongly associated with stereotyped locomotor patterns and postural control that have relatively consistent functional interpretations across observers. The addition of object contact (e.g., wiping a foot on a mat) may not substantially reduce interpretive ambiguity beyond what is already established by the locomotor context.

The overall pattern of stimulus-driven neural synchronization, including the key finding of superior ISC for leg movements, was successfully replicated in data from the second viewing, confirming the robustness of these effects across repeated exposures. This replicability demonstrates that the observed patterns of neural synchrony reflect stable, stimulus-driven properties rather than transient or session-specific factors.

A complementary framework for understanding the enhanced ISC for leg movements comes from recent work by Karakose-Akbiyik et al. (2023), who demonstrated that frontoparietal and posterior temporal regions encode a shared neural code for the physics of events, invariant to whether the agent is human or inanimate object. We propose that leg movements, being more stereotyped and biomechanically constrained (e.g., stepping, kicking, wiping), may more consistently and robustly engage this universal event-physics code across observers. In contrast, upper-limb movements exhibit immense kinematic diversity and are laden with context-dependent goals, which may recruit additional agent-specific systems—particularly in right pSTS/TPJ and bilateral SPL (Karakose-Akbiyik et al., 2023)—that introduce interpretive variability across individuals. This differential engagement of shared event-physics versus agent-specific neural systems provides a mechanistic account of why lower-limb movements drive stronger neural synchronization across viewers, while upper-limb movements elicit more individualized processing patterns.

A content-specific repetition effect was also observed, whereby ISC significantly increased during the second viewing specifically for arm movements, indicating potential learning or anticipation mechanisms unique to upper-limb action observation. This selective enhancement for arm videos may reflect reduced interpretive uncertainty upon second exposure: having already seen the arm actions once, participants may have developed more consistent mental models or expectations about the movement sequences, leading to greater alignment in their neural responses. The absence of such repetition effects for leg and nature videos suggests that these categories may have been processed more uniformly even during initial viewing, leaving less room for learning-related enhancement.

The scalp topography of the inter-subject correlation revealed that synchronized neural activity was strongest over occipitotemporal and parietal regions. This pattern is consistent with our previous work (Ntoumanis et al., 2023b) and aligns well with the known involvement of these areas in the visual and semantic processing of naturalistic stimuli. Occipitotemporal regions, including the superior temporal sulcus, are central to biological motion perception and the extraction of kinematic information from observed actions. Parietal regions, particularly areas near sensorimotor cortex, are implicated in motor resonance and the translation of observed actions into one’s own motor repertoire.

Interestingly, the first correlated component (C1) showed a predominantly frontal topography, possibly reflecting engagement of higher-level attentional, executive, or semantic processes that are time-locked across viewers as they interpret and make sense of observed actions. The prominence of frontal areas in C1, rather than the visual areas that typically dominate ISC in more passive viewing paradigms, may reflect the goal-directed, motor-perceptual nature of our task, where participants actively processed action-relevant information rather than simply registering visual features.

### Dissociation between ISC and IaSC

One of the most intriguing results of this study is the observed dissociation between the mechanisms governing neural alignment across individuals (ISC) and temporal consistency within an individual (IaSC). The striking similarity between the scalp topographies of the dominant ISC and IaSC components suggests that a common, distributed cortical network—encompassing high-level visual, frontal (attentional), and parietal (potentially motor-resonant) areas—supports both shared engagement across viewers and reliable processing within an individual over time (Dmochowski et al., 2012; Ntoumanis et al., 2023b).

However, a critical divergence emerged in how stimulus features influenced these measures. While object interaction and POV angle were significant modulators of ISC, they had no significant effect on IaSC magnitude. This indicates that these content characteristics are crucial for driving shared engagement across a group but are less critical for an individual’s neural consistency across viewings. This pattern implies that IaSC might be driven more by stable, trait-like individual differences in neural processing architecture than by transient, stimulus-driven properties. The core finding that IaSC was generally higher than ISC robustly supports the intuitive notion that an individual’s neural response is more similar to itself across time than to the responses of others, confirming the methodological validity of the IaSC approach. This dissociation provides a more nuanced understanding of brain dynamics during naturalistic vision, suggesting that ISC and IaSC tap into different neural and cognitive phenomena—one reflecting common, stimulus-driven engagement, and the other reflecting stable, individual-specific processing traits. This framework opens new avenues for research aiming to disentangle how individual differences and stimulus properties interact to shape perception.

Importantly, the present study employed a rigorously controlled design to isolate the effects of specific kinematic and contextual features on neural synchrony. This approach yielded results that refine our previous findings (Ntoumanis et al., 2021b). Specifically, the clear superiority of leg over arm movements in driving ISC was not observed in our earlier work. We posit that this discrepancy underscores the critical influence of methodology. The previous study used ecologically rich but complex video blocks where movements were embedded in varying contexts, and ISC was calculated in brief time-locked epochs. In contrast, the current paradigm utilized discrete, well-defined clips with a global ISC value calculated over the entire viewing duration. This approach provides a more stable measure of overall attentional engagement, mitigating potential contamination from unrelated contextual content.

Furthermore, the finding that object interaction is a significant moderator of ISC specifically for arm actions introduces a crucial nuance, suggesting that goal-directedness of an action is a critical factor – a dimension not captured by previous kinematic annotations. The successful replication of core results during a second viewing strengthens the conclusion that the effects are robust and stimulus-driven. To our knowledge, this is one of the first studies to measure IaSC using Correlated Component Analysis (CorrCA) for EEG in the context of naturalistic action perception, providing a novel window into the reliability of individual neural processing and its relationship to group-level synchronization.

### Limitations

Several limitations of this study should be acknowledged. First, while the sample size (*N* = 28) is consistent with similar EEG-ISC studies, a larger cohort would provide greater statistical power for exploring potential individual differences and more complex interactions. Second, the use of surface EEG, while excellent for capturing synchronized cortical activity with high temporal precision, limits our ability to make definitive claims about the specific contributions of deep brain structures (e.g., basal ganglia, hippocampus) to the observed effects. Third, despite meticulous control over filming conditions and the use of ALD as a covariate, it is inherently challenging to fully equate all perceptual features across distinct stimulus categories. Future studies employing complementary neuroimaging methods (e.g., fMRI for spatial resolution, MEG for source localization) would help address these limitations.

Two limitations related to stimulus construction warrant consideration. First, limb type was confounded with actor identity: all arm movements were performed by one adolescent actor, and all leg movements by another. Consequently, the observed ISC differences between arm and leg videos could theoretically reflect actor-specific factors (e.g., individual kinematic style, movement smoothness) rather than limb-specific affordances. To mitigate this concern, we conducted a per-video ISC analysis, which revealed consistent ISC values across videos within each limb category, suggesting that the limb-related pattern is not driven by idiosyncratic features of a single video or actor. Nonetheless, future studies should employ multiple actors per limb type to disentangle limb-specific from actor-specific effects, and our claims regarding limb specificity should be interpreted with appropriate caution. Second, the actors were children (ages 11–12) while our participants were adults (ages 18–34). Prior research indicates that neural responses during action observation can be tuned to age-congruent actors (Lui et al., 2011), potentially modulating the strength of motor resonance. While this age mismatch applies equally to both arm and leg conditions and thus does not compromise the comparative pattern between them, it may have affected absolute ISC magnitudes and could interact with limb type in ways our design cannot assess. Future work using age-matched stimuli would clarify whether the observed limb-related effects generalize across actor age.

## Conclusion

In summary, we demonstrate that observation of human actions, particularly lower-limb movements, elicits robust and replicable neural synchrony across viewers, which is further modulated by goal-directedness and perspective. The dissociation between ISC and IaSC reveals a key insight: while similar neural networks are engaged, the factors influencing shared group experiences and individual reliability are distinct. The present findings have potential clinical implications, as understanding the neural mechanisms underlying action perception and the dissociation between shared and individual neural responses could inform interventions for disorders characterized by deficits in social cognition and motor processing—such as autism spectrum disorder, stroke rehabilitation, and Parkinson’s disease—by targeting specific neural networks to enhance neural synchrony and improve social and motor functioning. This framework opens new avenues for research aiming to disentangle how individual differences and stimulus properties interact to shape perception in both typical and atypical populations.

The present findings offer several concrete avenues for clinical translation. In autism spectrum disorder, where deficits in social cognition and action understanding are well-documented, our stimulus set could be used to probe specific impairments in processing goal-directed actions versus non-biological motion, with ISC reductions serving as a potential neural marker of social attention dysfunction. For stroke rehabilitation, particularly in patients with upper-limb motor impairments, the limb-specific modulation of neural synchrony by object interaction and perspective suggests that observing well-controlled action videos—especially those depicting manipulable objects from a first-person perspective—could enhance motor resonance and support recovery of function when integrated into action observation therapy protocols. In Parkinson’s disease, where basal ganglia dysfunction impairs rhythmic movement initiation and execution, the metronome-paced leg movement stimuli might be adapted to assess or train neural entrainment to locomotor rhythms, potentially informing gait rehabilitation strategies (Hackney et al., 2015). The dissociation between ISC and IaSC further suggests that clinical assessments could separately probe shared neural engagement (ISC, reflecting social attunement) and individual processing stability (IaSC, reflecting consistency of neural function), offering complementary metrics for evaluating intervention efficacy and tracking recovery across different patient populations.

## Data Availability

The datasets presented in this article are not readily available because the datasets generated and analyzed for this study will be made available by the corresponding author upon reasonable request. Requests to access the datasets should be directed to Maria Koriakina, mkoriakina@hse.ru.

## Footnotes

1
https://free-stock.videos/
