# Supplementary figures and images for: Validation of novel naturalistic limb movement stimuli for studying biological motion perception in adults
Source: Front Hum Neurosci. 2026 May 7;20:1754368. doi: 10.3389/fnhum.2026.1754368 (PMC13190463; doi:10.3389/fnhum.2026.1754368)

## A Experimental design

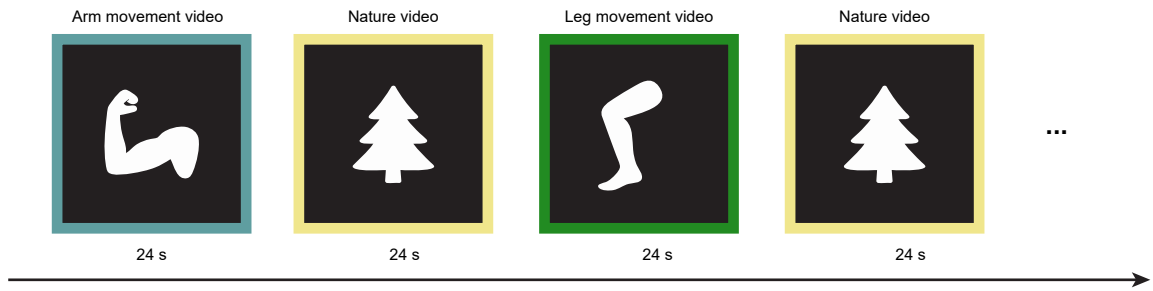

## B Factors of the arm/leg movement videos

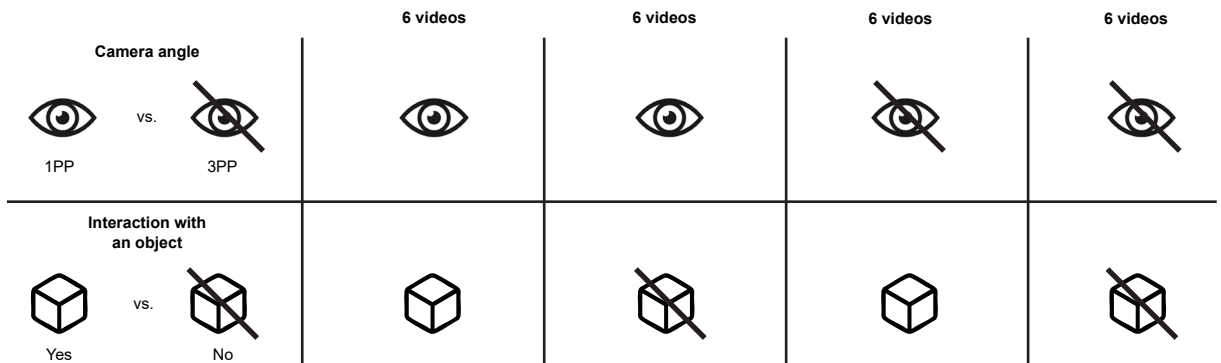

## C Data analysis pipeline

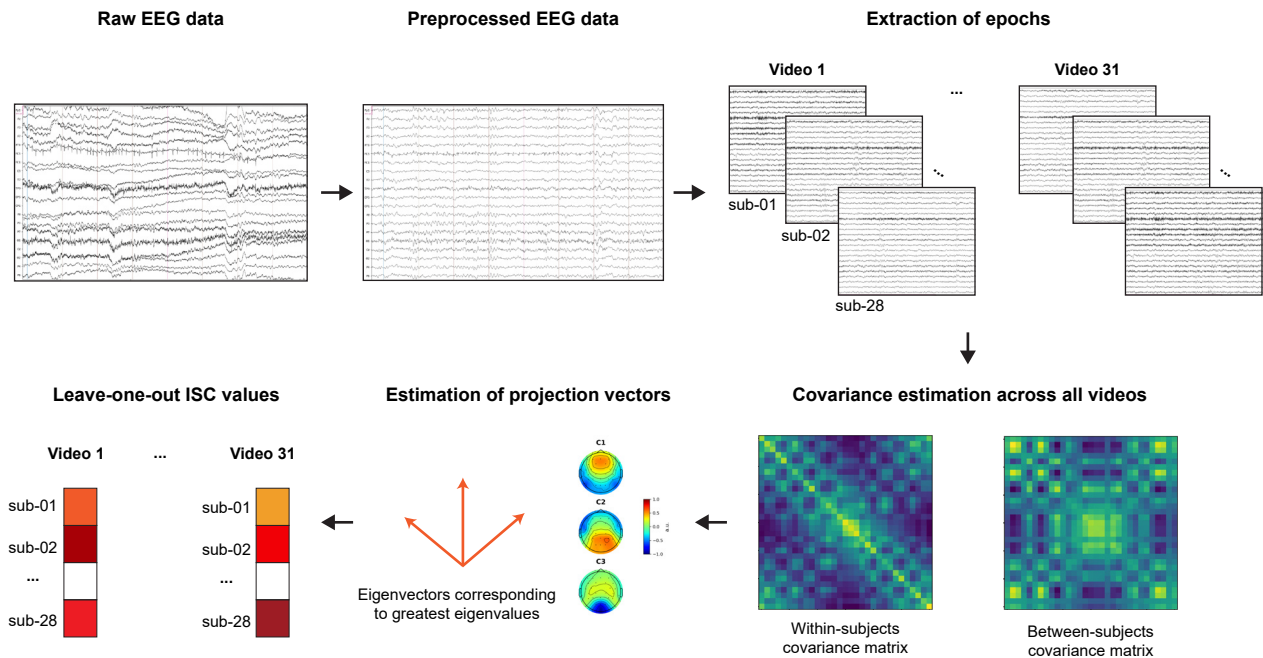

Supplement: Supplementary file 1 [file Data_Sheet_1.pdf]
