# Supplementary material for: Validation of novel naturalistic limb movement stimuli for studying biological motion perception in adults
Source: Front Hum Neurosci. 2026 May 7;20:1754368. doi: 10.3389/fnhum.2026.1754368 (PMC13190463; doi:10.3389/fnhum.2026.1754368)
Supplement: Supplementary file 2 [file Data_Sheet_2.docx]

# Supplementary Materials

**Supplementary Table 1**

*Set of stimuli used in the study. The video name matches the filename in the online repository with the stimuli. The condition indicates whether each video featured arm movement, leg movement, or nature scenes. The arm and leg movement videos are further described by whether or not they involved interaction with an object and by whether or not they were recorded with a POV camera angle.*

| Video name | Condition | Interaction with  an object | POV camera angle |
| --- | --- | --- | --- |
| 1AO1 | Arm | Yes | Yes |
| 1AO2 | Arm | Yes | Yes |
| 1AO3 | Arm | Yes | Yes |
| 1AN1 | Arm | No | Yes |
| 1AN2 | Arm | No | Yes |
| 1AN3 | Arm | No | Yes |
| 2AO1 | Arm | Yes | No |
| 2AO2 | Arm | Yes | No |
| 2AO3 | Arm | Yes | No |
| 2AN1 | Arm | No | No |
| 2AN2 | Arm | No | No |
| 2AN3 | Arm | No | No |
| 1LN1 | Leg | No | Yes |
| 1LN2 | Leg | No | Yes |
| 1LN3 | Leg | No | Yes |
| 1LO1 | Leg | Yes | Yes |
| 1LO2 | Leg | Yes | Yes |
| 1LO3 | Leg | Yes | Yes |
| 2LN1 | Leg | No | No |
| 2LN2 | Leg | No | No |
| 2LN3 | Leg | No | No |
| 2LO1 | Leg | Yes | No |
| 2LO2 | Leg | Yes | No |
| 2LO3 | Leg | Yes | No |
| NAT1 | Nature | N/A | N/A |
| NAT2 | Nature | N/A | N/A |
| NAT3 | Nature | N/A | N/A |
| NAT4 | Nature | N/A | N/A |
| NAT5 | Nature | N/A | N/A |
| NAT6 | Nature | N/A | N/A |
| NAT7 | Nature | N/A | N/A |

**Supplementary Table 2**

*Results of the control analysis conducted to verify the frequency of each oddball category was similar across the video conditions. Each cell indicates the average number of standard (Std) and deviant (Dev) words presented in each pair of conditions. For example, while viewing arm movement videos, participants listened to 17.86 standard words related to leg movement, on average.*

| Video condition | Arm movement- related words  (Std/Dev) | Leg movement- related words  (Std/Dev) | Neutral / pseudowords  (Std/Dev) |
| --- | --- | --- | --- |
| Arm | 18.22 / 4.80 | 17.86 / 4.84 | 17.96 / 4.67 |
| Leg | 17.97 / 4.74 | 18.15 / 4.85 | 18.31 / 4.69 |
| Nature | 18.23 / 4.78 | 18.11 / 4.89 | 18.31 / 4.69 |


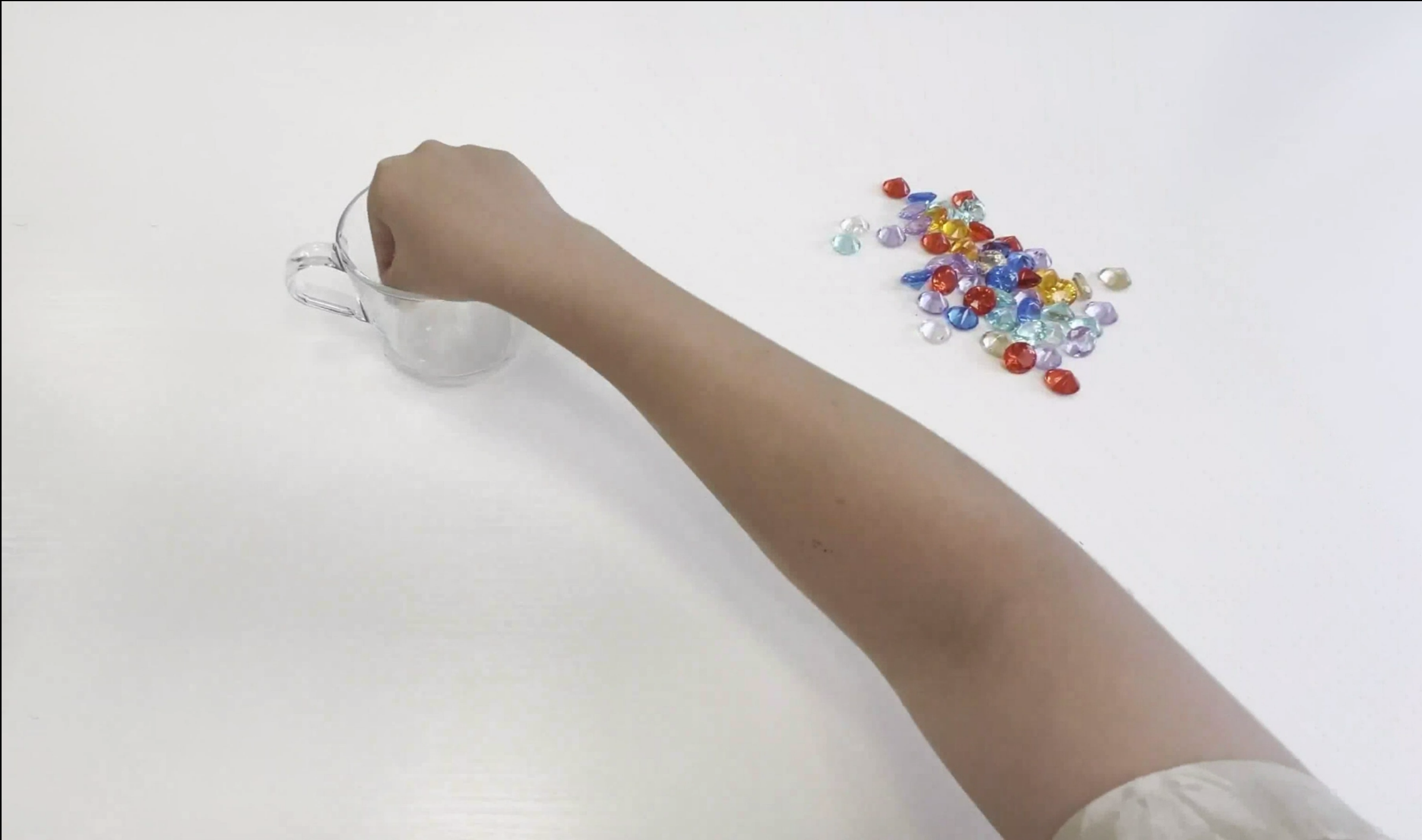
 **Supplementary materials S1.** Screenshot of the video stimulus: viewpoint - first-person, limb – upper, object presence – with object.
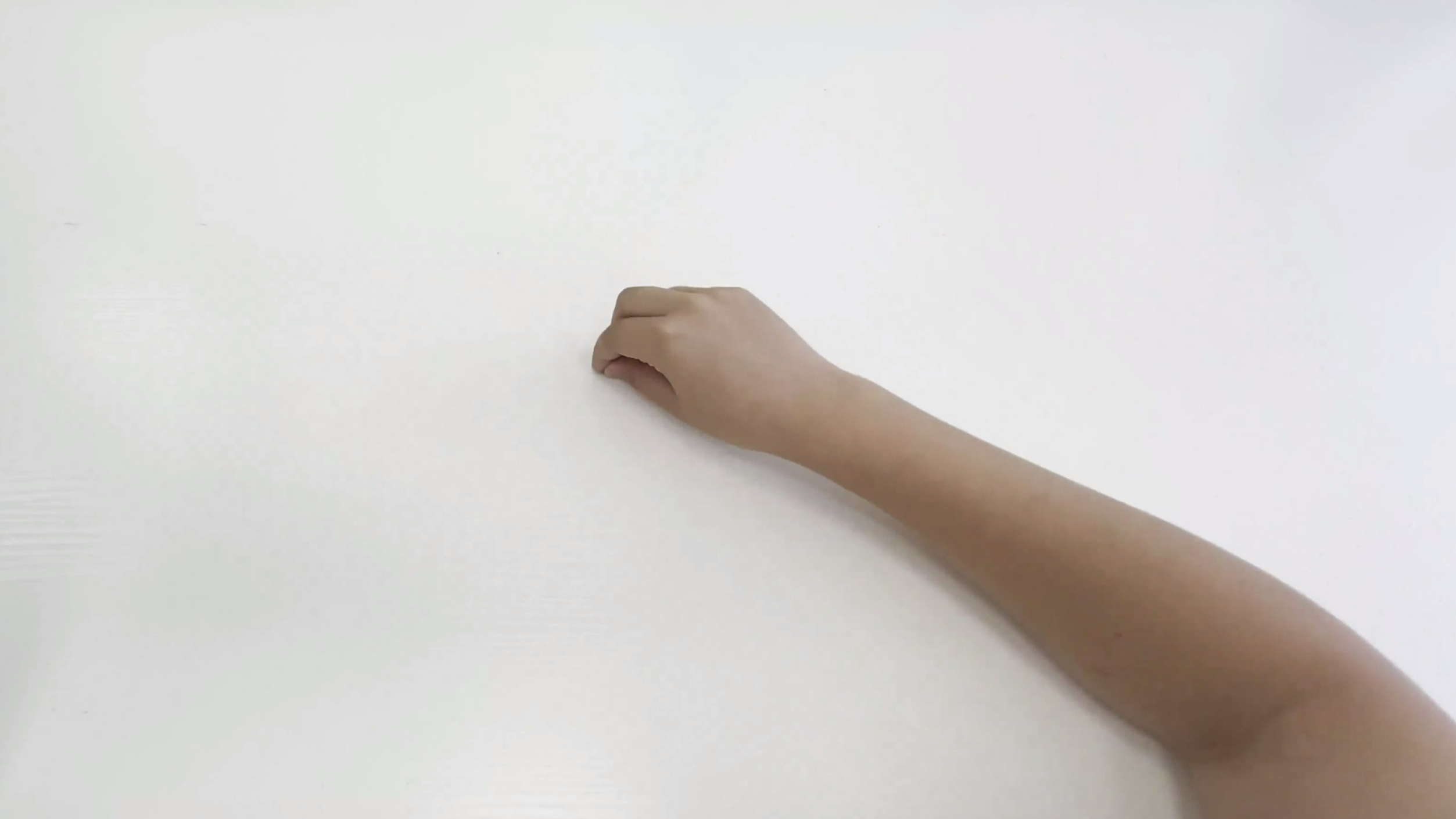


**Supplementary materials S2.** Screenshot of the video stimulus: viewpoint - first-person, limb – upper, object presence – no object.


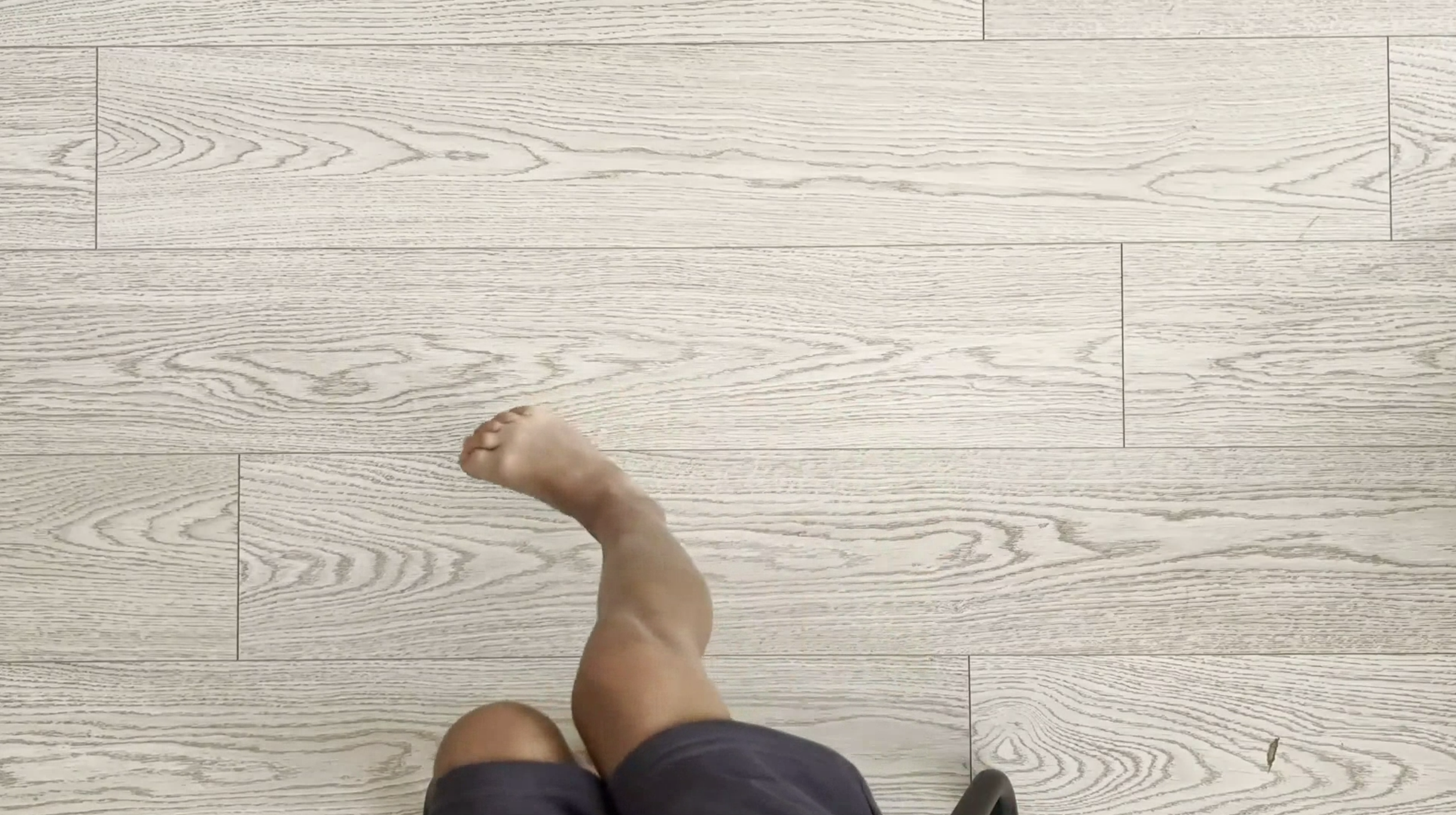


**Supplementary materials S3.** Screenshot of the video stimulus: viewpoint - first-person, limb – lower, object presence – no object.


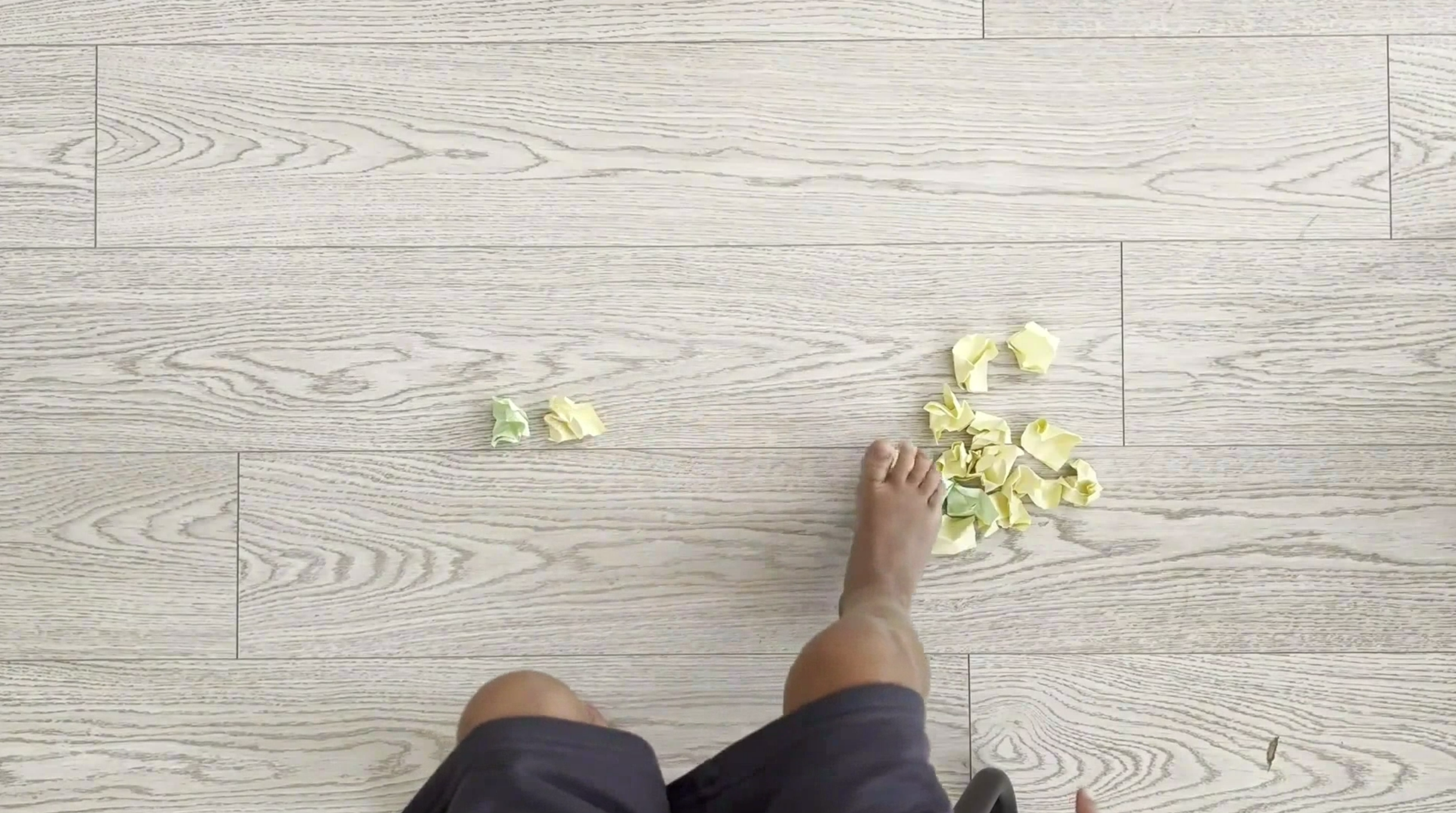


**Supplementary materials S4.** Screenshot of the video stimulus: viewpoint - first-person, limb – lower, object presence – with object.


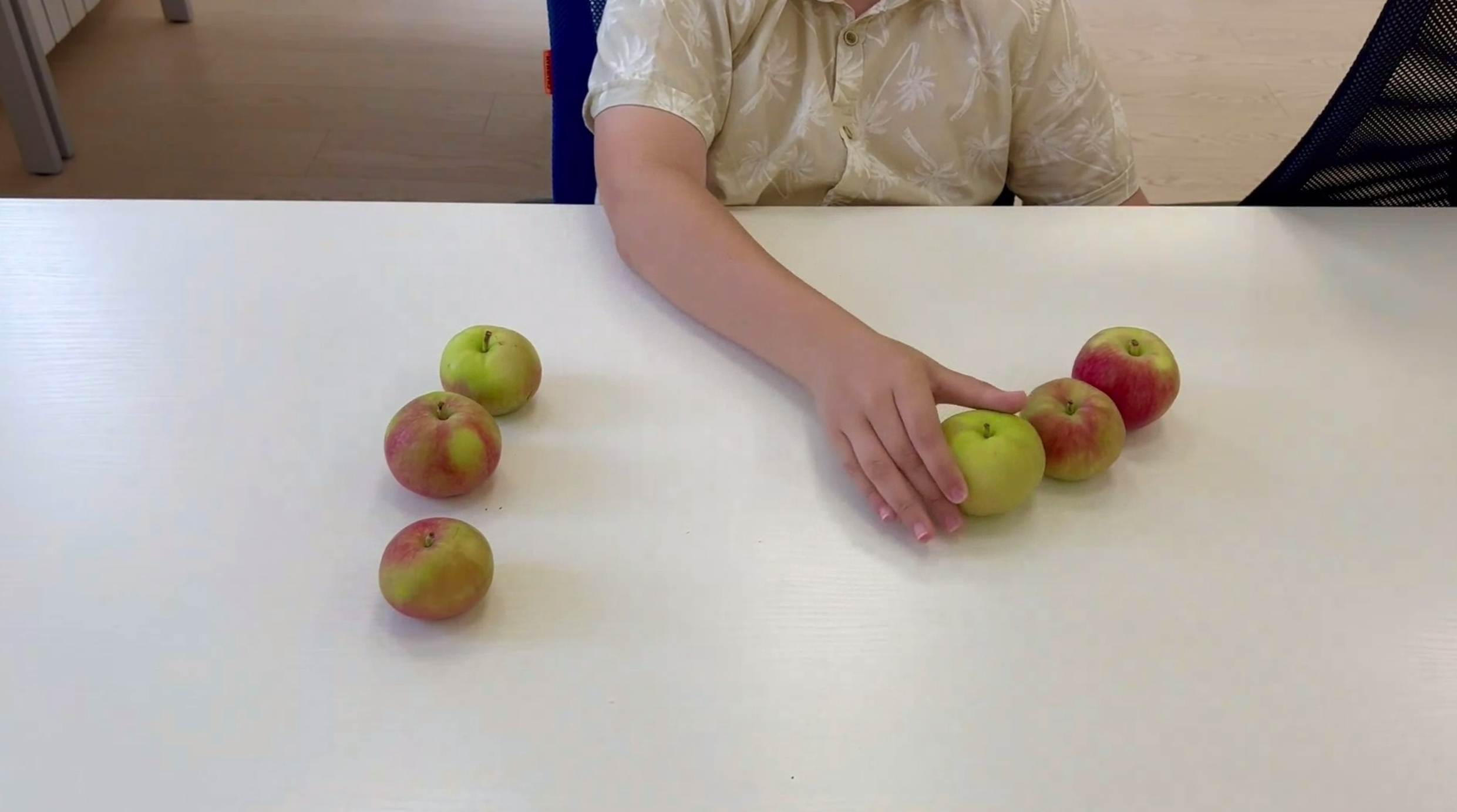


**Supplementary materials S5.** Screenshot of the video stimulus: viewpoint – frontal perspective, limb – upper, object presence – with object.


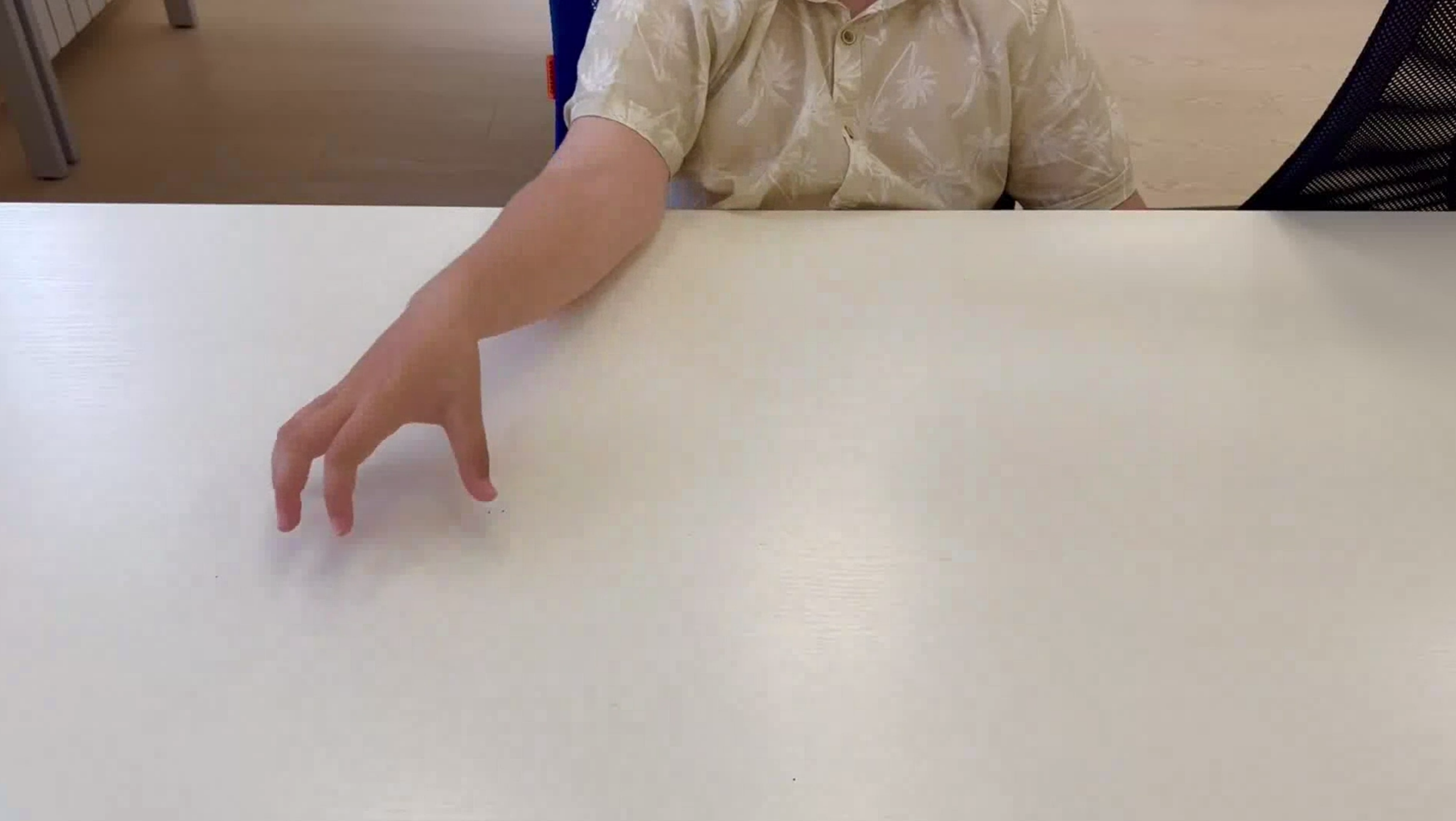


**Supplementary materials S6.** Screenshot of the video stimulus: viewpoint – frontal perspective, limb – upper, object presence – no object.


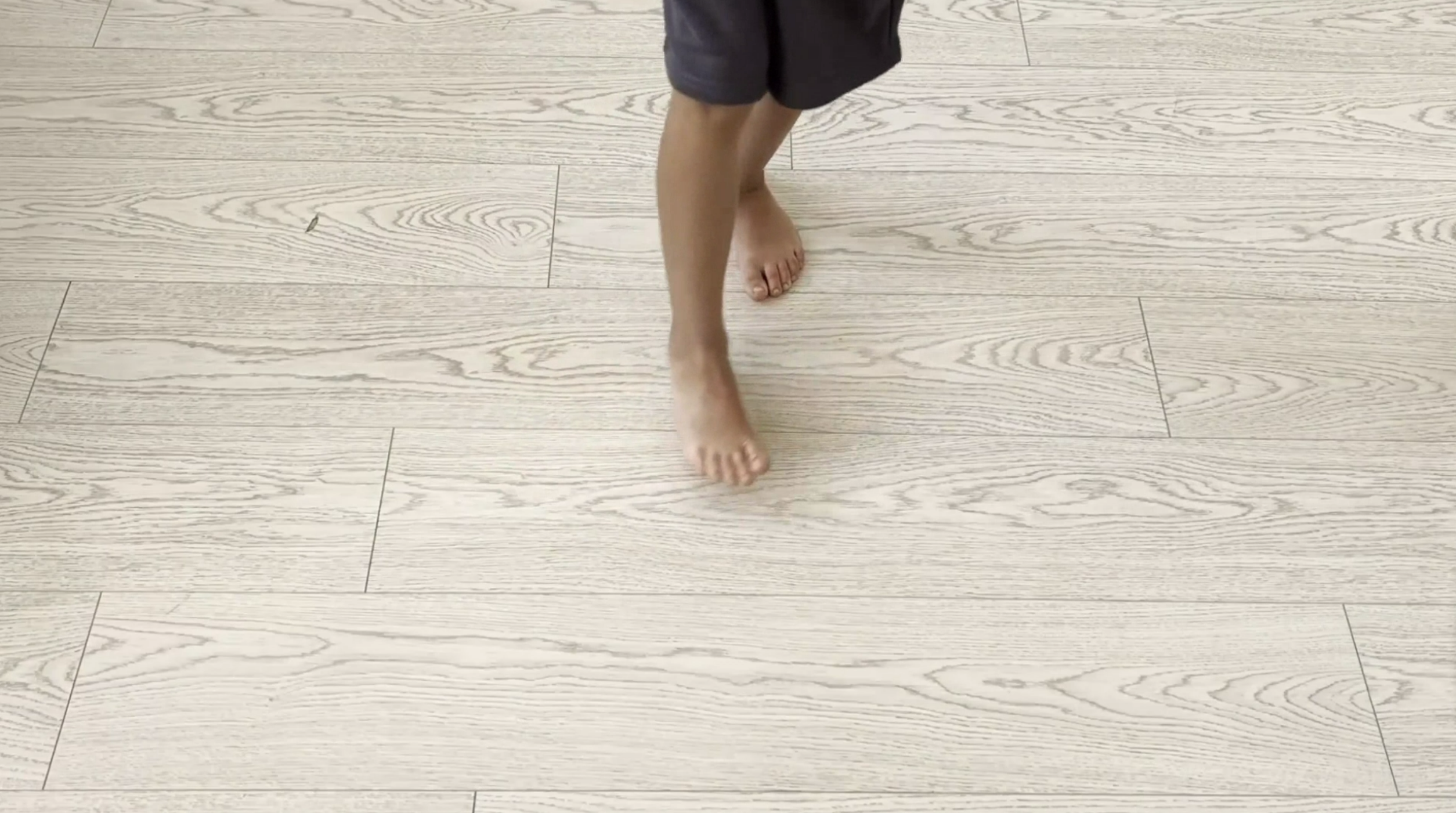


**Supplementary materials S7.** Screenshot of the video stimulus: viewpoint – frontal perspective, limb – lower, object presence – no object.


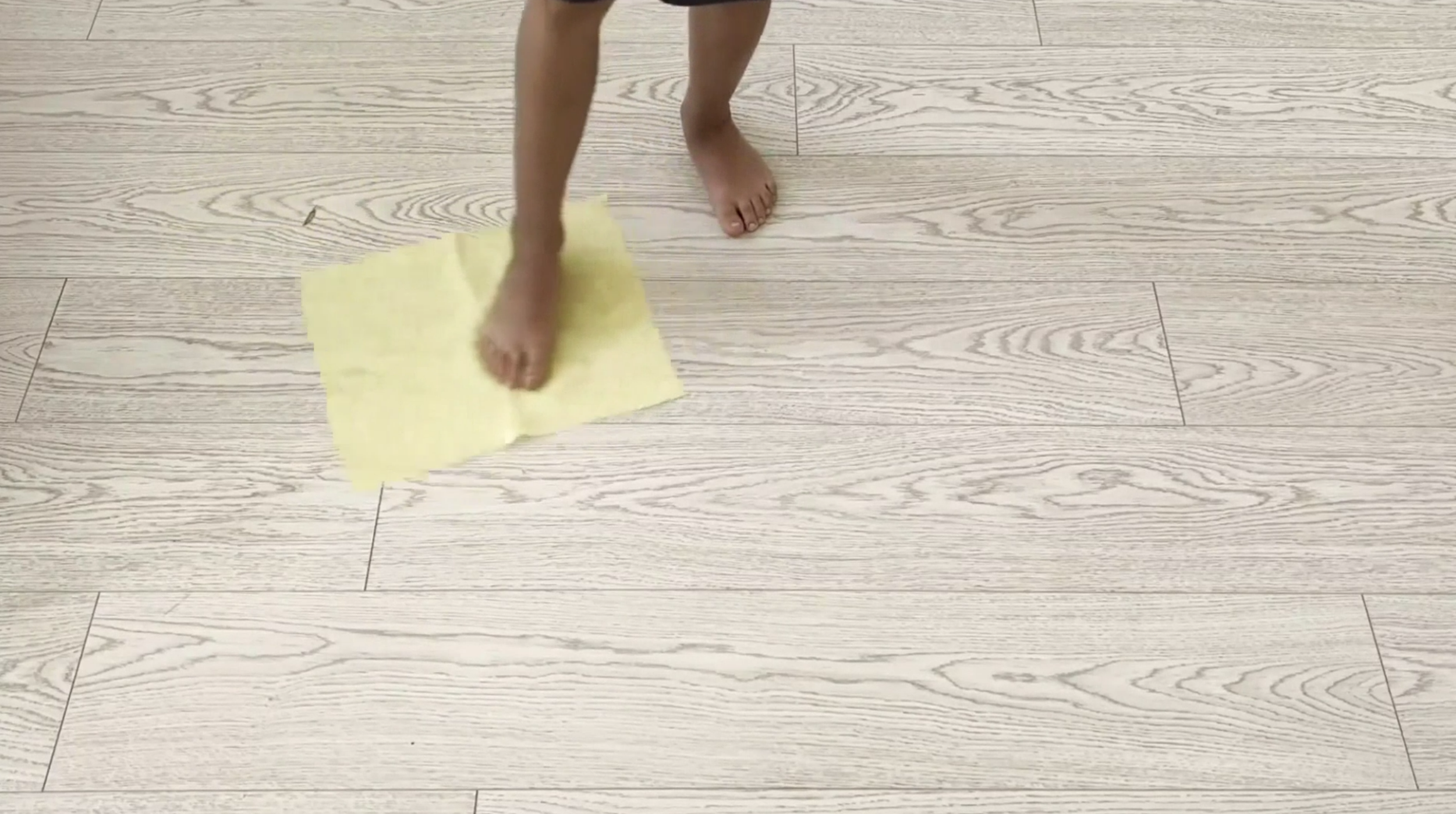


**Supplementary materials S8.** Screenshot of the video stimulus: viewpoint – frontal perspective, limb – lower, object presence – with object.
